# Mitochondrial stress-signaling networks in male reproductive dysfunction: redox, inflammatory, and cell-death integration with context-dependent modulation by melatonin

**DOI:** 10.3389/fphys.2026.1990053

**Published:** 2026-10-28

**Authors:** Qi Qi, Jingsheng Luo, Xuejiao Ren, Lin Jiang, Tianhui Liu, Wei Liu, ChangJie Bao, Meiyan Sun, Hongxun Fu

**Affiliations:** 1 College of Laboratory Medicine, Jilin Medical University, Jilin, China; 2 School of Life Science and Technology, Changchun University of Science and Technology, Changchun, China; 3 College of Biomedical Engineering, Jilin Medical University, Jilin, China

**Keywords:** DNA integrity, environmental toxicants, male reproductive dysfunction, melatonin, mitochondrial dysfunction, oxidative stress, regulated cell death

## Abstract

Mitochondria integrate bioenergetic, redox, calcium, inflammatory, and cell-death signals during cellular stress, but their position within male reproductive injury is highly dependent on cell type and initiating insult. This critical narrative review examines mitochondrial stress-signaling networks in Leydig cells, Sertoli cells, developing germ cells, and spermatozoa, with particular attention to their context-dependent modulation by melatonin. Physiological reactive oxygen species (ROS) participate in steroidogenesis and sperm function, whereas excessive or mislocalized ROS can interact bidirectionally with mitochondrial dysfunction, inflammatory signaling, impaired quality control, and regulated cell death. Across toxicant, metabolic, inflammatory, thermal, ischemic, aging, and cryopreservation models, mitochondria can therefore function as conditional integrative signaling hubs rather than universal initiating causes. Melatonin-associated responses intersect with these networks through MT1/MT2 signaling, redox-sensitive processes, chemical interactions with reactive species, and mitochondria-associated mechanisms, with reported effects on mitochondrial function, inflammatory amplification, apoptosis, and ferroptosis- or pyroptosis-associated injury. However, molecular co-variation is distinguished from causal signaling, and lipid peroxidation or inflammasome activation alone is not considered definitive evidence of ferroptosis or pyroptosis. Human evidence remains limited relative to animal, ex vivo, and cell-culture studies. We propose an evidence-calibrated initiation–amplification–progression framework in which mitochondria integrate stress signals dynamically and melatonin acts as a context-dependent experimental modulator rather than an established reproductive therapy.

## 1 Introduction

Male factor infertility is a multifactorial condition involving genetic, endocrine, anatomical, infectious, metabolic, environmental, pharmacological, and lifestyle factors (Agarwal et al., 2021; Minhas et al., 2025; Zhao et al., 2025). No single pathway, including mitochondrial dysfunction, accounts for all cases. Reactive oxygen species (ROS) illustrate this complexity: spatially and temporally controlled ROS participate in steroidogenesis, sperm maturation, capacitation, hyperactivation, the acrosome reaction, and sperm–oocyte interaction (Agarwal et al., 2003; Aitken and Curry, 2011; Agarwal et al., 2014; Aitken et al., 2016), whereas excessive, persistent, or mislocalized ROS can overwhelm antioxidant and repair systems and promote cellular injury. Thus, the relevant biological issue is not whether ROS are uniformly beneficial or harmful, but how reproductive cells maintain an appropriate redox range under physiological and pathological conditions. Mitochondrial metabolism, oxidative phosphorylation, calcium handling, and redox signaling contribute to reproductive-cell function to different extents according to cell type, species, and physiological state (Brookes et al., 2004; Murphy, 2009; Amaral et al., 2013; Vertika et al., 2020; Park and Pang, 2021).

Oxidative stress can promote membrane dysfunction, protein oxidation, lipid peroxidation, DNA damage, and altered signaling or gene expression (Ooe et al., 2005; Yang et al., 2023; Sengupta et al., 2024; Chen et al., 2025; Wang Y. et al., 2025). However, reproductive injury is not organized around a single obligatory sequence. Aging, circadian disruption, toxicant exposure, metabolic disease, dysbiosis, inflammation, and other insults may disturb redox balance through different initiating events (Xu Y. et al., 2020; Wang et al., 2022; Zhang Z. et al., 2024; Boeri et al., 2025; Kaltsas et al., 2025; Zhao et al., 2025). Mitochondrial injury may occur early in some models but may follow endocrine disturbance, receptor dysregulation, inflammation, genotoxicity, or junctional dysfunction in others. This distinction is particularly important in the male reproductive system because Leydig cells, Sertoli cells, developing germ cells, and spermatozoa have different metabolic and mitochondrial requirements. Leydig-cell mitochondria participate directly in steroidogenesis (Payne and Hales, 2004; Miller and Auchus, 2011; Zirkin and Papadopoulos, 2018), Sertoli cells integrate mitochondrial function with glycolytic support, immune regulation, and blood–testis barrier maintenance (Oliveira et al., 2015; Feng et al., 2022; Kabbesh et al., 2022), germ cells undergo stage-dependent metabolic remodeling, and sperm bioenergetics vary among species and experimental conditions (Abruzzese et al., 2024; Meng et al., 2026; Rong et al., 2026).

Mitochondria nevertheless occupy an important intersection among bioenergetics, calcium handling, redox signaling, innate immunity, and regulated cell death (Murphy, 2009; Amaral et al., 2013; Bock and Tait, 2020; Abruzzese et al., 2024; Bravo et al., 2024). Mitochondrial ROS, membrane depolarization, cytochrome c release, mitochondrial DNA, and cardiolipin redistribution may contribute to inflammatory and cell-death-associated signaling, while inflammatory signaling can in turn impair mitochondrial function (Jou et al., 2007; Morgan and Liu, 2011; Shimada et al., 2012; Chen et al., 2020; Miao et al., 2022; Wu et al., 2024; Yu et al., 2024; Zhang Z. et al., 2024; Dai et al., 2026). Melatonin is particularly relevant to this network because its reported reproductive actions intersect with several of these mechanisms rather than with a single antioxidant pathway. In addition to signaling through the established MT1 and MT2 receptors, melatonin can participate in circadian regulation, redox-sensitive signaling, chemical reactions with reactive oxygen and nitrogen species, and mitochondria-associated processes (Reiter, 1991; Gonzalez-Arto et al., 2016; Perumal et al., 2020; Hassan et al., 2022; Jauhari et al., 2025). Experimental studies have associated melatonin exposure with preservation of mitochondrial membrane potential and bioenergetics, attenuation of excessive ROS accumulation, modulation of inflammatory signaling, and reduced cell-death-associated injury in reproductive models (Bahrami et al., 2018; Baş and Nazıroğlu, 2019; Wang et al., 2021; Niu et al., 2025). These observations provide a mechanistic rationale for examining melatonin together with mitochondrial stress signaling; however, they do not establish that mitochondrial protection is always the initiating mechanism or that melatonin acts through one conserved pathway across reproductive cell types.

Important mechanistic and translational uncertainties remain. Mitochondrial accumulation of melatonin is supported (Venegas et al., 2012; Reiter et al., 2017; Reiter et al., 2020; Tan et al., 2023), whereas autonomous melatonin synthesis in male reproductive mitochondria has not been directly demonstrated and remains an extrapolation from neuronal and oocyte studies (He et al., 2016; Suofu et al., 2017; Jauhari et al., 2020; Zhu et al., 2024). MT1 and MT2 are established G-protein-coupled melatonin receptors (Dubocovich et al., 2010), whereas retinoic acid receptor-related orphan receptor α (RORα) is better considered a possible downstream or parallel circadian transcriptional regulator; direct physiological melatonin–RORα binding remains controversial (Coelho et al., 2019; Li et al., 2020). Moreover, most protective evidence derives from rodents, livestock, reproductive-cell cultures, or ex vivo systems, whereas human evidence remains limited (Anderson et al., 1993; Espino et al., 2010; Bahrami et al., 2018; Chumboatong et al., 2020; Zhao et al., 2021; Feng et al., 2022; Zhang et al., 2022; Sahu and Verma, 2023; Chen et al., 2024). These limitations require a clear distinction between pathway association and causality and between preclinical biological plausibility and demonstrated clinical reproductive efficacy.

The distinctive aim of this critical narrative review is to integrate the melatonin literature within a cell-specific and evidence-calibrated model of male reproductive stress signaling. Rather than presenting melatonin as a generic antioxidant or assembling individual signaling pathways into a universal linear cascade, we distinguish physiological from pathological ROS signaling, examine mitochondria as context-dependent integrative nodes, separate mechanistic association from stronger causal evidence, and differentiate human, animal, ex vivo, and cell-culture findings. We further synthesize the available evidence into a proposed initiation–amplification–progression framework linking mitochondrial stress, inflammatory signaling, regulated cell-death-associated injury, and loss of reproductive function. Throughout the review, melatonin is discussed as a context-dependent experimental modulator rather than as an established therapy.

## 2 Narrative review approach

This critical narrative review was informed by targeted searches of PubMed, Web of Science Core Collection, and Scopus from database inception to 31 July 2026. The searches combined concepts related to melatonin, male reproductive tissues and dysfunction, redox and mitochondrial stress, inflammatory signaling, and regulated cell death. No language, species, or study-design restrictions were applied at the database-search stage. Human studies and samples, animal models, ex vivo reproductive material, and reproductive-cell systems were prioritized when they provided mechanistic or functional information relevant to the review questions. Studies published within the preceding 10 years were prioritized for contemporary evidence, whereas earlier seminal or foundational studies were retained when necessary; this interval was not applied as an absolute exclusion criterion. Duplicate reports, abstracts lacking sufficient experimental information, studies without male reproductive relevance, and indirect non-reproductive studies without a clear mechanistic role in the discussion were not treated as primary reproductive evidence. After database records were merged, candidate records were evaluated narratively for their relevance to the scope and evidence framework of this review, with targeted citation checking used when necessary to identify seminal or supporting literature. Literature identification and selection were undertaken to support a critical narrative synthesis rather than a systematic review or meta-analysis; therefore, no formal risk-of-bias assessment or quantitative evidence synthesis was performed. For transparency, the database-specific search strategies, final search dates, search yields, evidence-selection process, and classification of the final cited evidence set are provided in **Supplementary Table S1**.

## 3 Cell-specific redox vulnerability and melatonin-responsive context

The hypothalamic-pituitary-gonadal axis, alongside Leydig and Sertoli cells, developing germ cells, sperm, immune cells and the epididymal microenvironment, are all required for normal functioning of the male reproductive system (Rato et al., 2012; Tremblay, 2015; Zirkin and Papadopoulos, 2018; Fusco et al., 2021; Kabbesh et al., 2022; Zhai et al., 2024). These populations have specific and differential roles for mitochondria. For instance, Leydig cells utilize mitochondria for steroidogenesis, while these organelles are integrated with glycolysis and lactate in Sertoli cells. Germ cells restructure mitochondrial metabolism during their differentiation, and heterogeneity exists in the function of the mitochondrial sheath that encapsulates sperm of various species (Boussouar and Benahmed, 2004; Rato et al., 2012; Oliveira et al., 2015; Zirkin and Papadopoulos, 2018; Zhao et al., 2025). Therefore, findings in a single cell or species most definitely cannot encompass the entire male reproductive tract.

### 3.1 Cell-specific sites of ROS initiation and injury

#### 3.1.1 Physiological and pathological ROS signaling

ROS are often described as damaging byproducts of aerobic metabolism. This is oversimplified. At low concentrations and in a spatially regulated manner, ROS act as signaling molecules that modify other proteins with a redox-sensitive function and play a role in normal cellular adaptation (Anderson et al., 1993; Serafini and O'Flaherty, 2022). The production of mitochondrial ROS is interrelated with other factors including the electron transport chain, calcium, available nutrients, and the cell’s energy demand (Brookes et al., 2004; Murphy, 2009). In the male reproductive system, ROS mediate various functions associated with steroidogenesis and post-testicular functions of sperm including capacitation, hyperactivation, tyrosine phosphorylation, acrosomal responsiveness, and interacting with the oocyte (Pasqualotto et al., 2000; Amaral et al., 2013; Ko et al., 2014; Barbagallo et al., 2020; Ribas-Maynou and Yeste, 2020). For this reason, the elimination of ROS is not a desirable goal of biological systems; rather, reproductive systems must control the amount, duration, spatial localization, and the types of targets where ROS are active. The transition from signaling to oxidative damage occurs when ROS production exceeds antioxidant and repair capacities, or when redox signals remain activated (Pintus and Ros-Santaella, 2021) (**Figure 1A**). Damaging ROS can oxidize membrane phospholipids, damage mitochondrial and nuclear DNA, alter the function of proteins, disrupt calcium homeostasis, and impair mitochondrial membrane potential (Brookes et al., 2004; Jauhari et al., 2020; Guo et al., 2023; Bravo et al., 2024). The sperm membrane is highly susceptible to oxidative damage due to its high content of polyunsaturated fatty acids (Lenzi et al., 1996). Spermatozoa also have a limited cytoplasm, a higher susceptibility to damage, and a restricted capacity for repair (Aitken and Drevet, 2020). There is a two-way relationship between ROS and mitochondrial dysfunction. Excessive electron leakage and ROS from mitochondria may cause further mitochondrial damage (Ooe et al., 2005; Morgan and Liu, 2011; Niu et al., 2025). Thus, mitochondrial ROS should not be the only contributor to oxidative stress in reproduction. Inflammation, peroxisomes, endoplasmic reticulum stress, and exogenous factors may also contribute (Fiorini et al., 2004; Yin et al., 2017; Kabel et al., 2022; Habiba et al., 2023).

**Figure 1:**
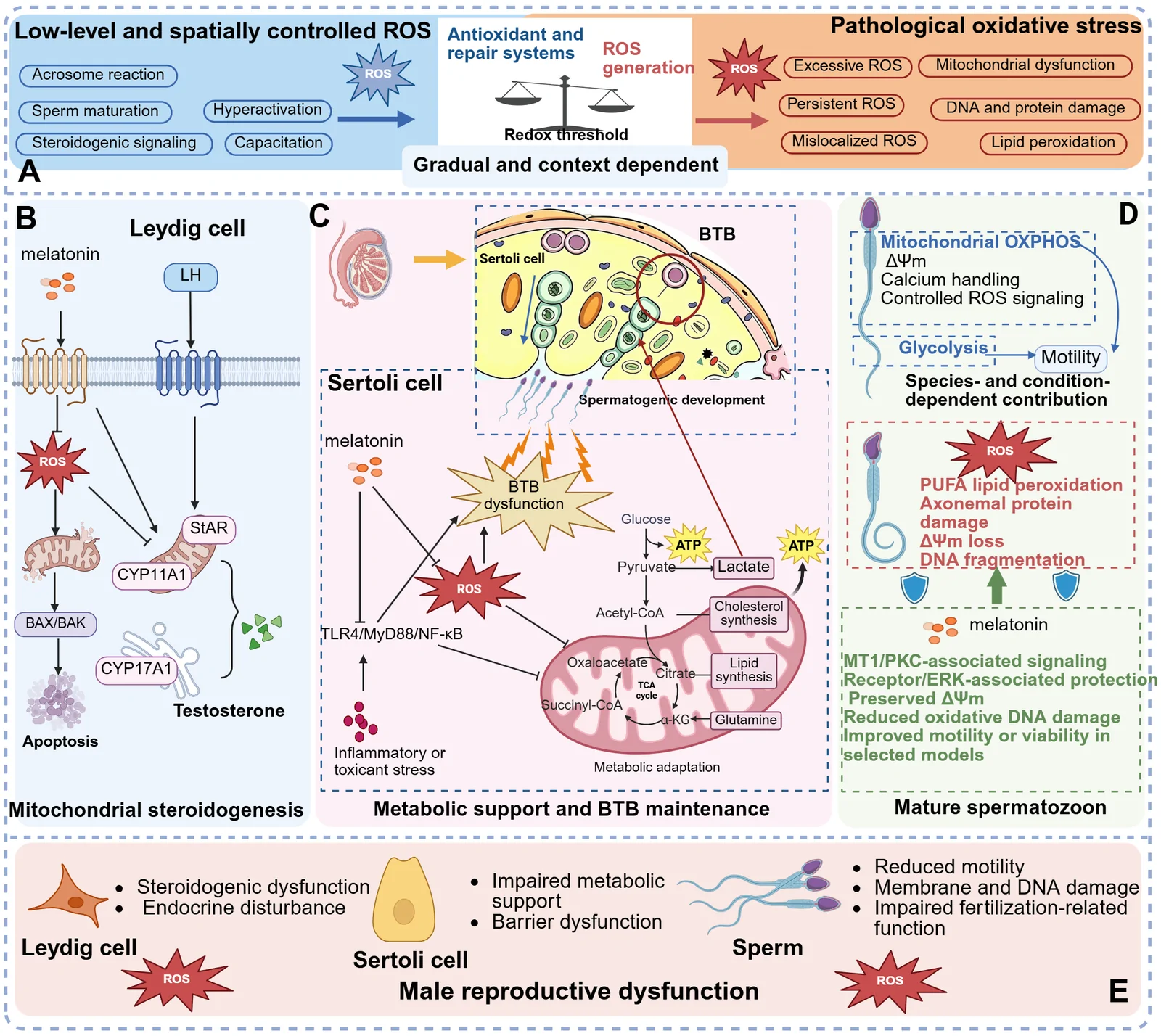
Cell-specific redox homeostasis, mitochondrial vulnerability, and melatonin-responsive mechanisms in the male reproductive system. **(A)** Physiological versus pathological ROS signaling. **(B)** Leydig-cell mitochondrial and steroidogenic responses. **(C)** Sertoli-cell metabolism, inflammatory signaling, and blood–testis barrier regulation. **(D)** Sperm bioenergetics, redox signaling, and mitochondrial injury. **(E)** Convergence of cell-specific injury on reproductive dysfunction. Melatonin-associated effects represent model-specific experimental observations and not established universal or clinical responses.

#### 3.1.2 Leydig cells: steroidogenesis and context-dependent responses

This is especially relevant when looking at melatonin. A decrease in a ROS marker that was elevated in the experiment may indicate the restoration of redox homeostasis, but it does not indicate that all ROS-dependent signaling has been inhibited or whether inhibition of such a process is beneficial. Leydig cells synthesize testosterone in the presence of luteinizing hormone and local regulatory signals (Fusco et al., 2021). Steroidogenesis starts with the movement of cholesterol from the outer to the inner mitochondrial membrane, and this occurs as a result of steroidogenic acute regulatory protein (Miller and Auchus, 2011). CYP11A1 subsequently converts cholesterol to pregnenolone, which is further metabolized by enzymes located in mitochondria and the smooth endoplasmic reticulum (Miller and Auchus, 2011). Thus, mitochondrial membrane integrity, cholesterol transport, redox state, and energy metabolism can influence Leydig-cell steroidogenic capacity. Mitochondrial dysfunction in Leydig cells may reduce steroidogenic acute regulatory protein activity, alter CYP11A1-dependent cholesterol conversion, increase ROS production, and promote apoptosis (Payne and Hales, 2004; Savchuk et al., 2013; Chen et al., 2019; Dai et al., 2026). Nevertheless, impaired testosterone production is not always initiated by mitochondrial injury. Reduced luteinizing hormone signaling, androgen receptor abnormalities, disrupted lipid-droplet metabolism, direct enzyme inhibition, inflammatory cytokines, or developmental defects may precede mitochondrial changes (Tremblay, 2015; Jin et al., 2024). Mitochondria are therefore one important component of Leydig-cell pathology rather than a universal initiating event. Experimental studies associate melatonin with Leydig-cell survival and steroidogenic responses together with MT1/MT2-related signaling, phosphoinositide 3-kinase/protein kinase B (PI3K/AKT) activity, sirtuin 1 (SIRT1)/peroxisome proliferator-activated receptor γ coactivator 1α (PGC-1α)-associated responses, and changes in steroidogenic proteins (Gao et al., 2019; Ma et al., 2021; Zhang et al., 2022; Pan et al., 2023; Chen et al., 2024; Liu et al., 2025). The strength of mechanistic inference differs among models. In mouse Leydig cells, depletion of melatonin receptors altered human chorionic gonadotropin-induced testosterone production, providing stronger receptor-level evidence (Gao et al., 2019), whereas changes in phosphorylation or protein abundance in other models primarily support pathway association. Melatonin has also been associated with protection against cisplatin-, phthalate-, toxin-, and metabolic-stress-related Leydig-cell injury (Bahrami et al., 2018; Yang et al., 2021; Zhang et al., 2022; Ma et al., 2023a; Chen et al., 2024). These effects are not uniformly stimulatory: melatonin inhibited testosterone synthesis in rooster Leydig cells in association with altered lipid-droplet lipolysis (Zhu et al., 2022), and responses vary with species, photoperiod, reproductive state, dose, timing, and receptor expression (Frungieri et al., 2005; Perumal et al., 2020; Xu et al., 2021; Hassan et al., 2022). Melatonin is therefore better described as a context-dependent modulator of steroidogenic function than as a universal testosterone enhancer (**Figure 1B**). Human endocrine evidence remains limited; oral melatonin was associated with changes in testicular inflammatory and oxidative markers in men with altered spermatogenesis (Riviere et al., 2020), without demonstrating improved fertility.

#### 3.1.3 Sertoli cells and blood–testis barrier integrity

Sertoli cells construct and support the seminiferous epithelium and build the blood-testis barrier. Further, Sertoli cells support developing germ cells by providing nutritional substrates as well as regulating the passage of developing germ cells (Cheng and Mruk, 2012). Their metabolic program differs from that of Leydig cells. Although Sertoli cells contain functionally important mitochondria, they also display pronounced glycolytic activity and convert glucose to lactate, which is used by developing germ cells (Boussouar and Benahmed, 2004; Rato et al., 2012; Alves et al., 2013; Oliveira et al., 2015). This metabolic arrangement means that impaired Sertoli-cell support cannot be explained solely by reduced mitochondrial adenosine triphosphate production. Blood–testis barrier dysfunction may result from altered tight-junction proteins, actin remodeling, androgen signaling, inflammatory activation, integrated stress responses, metabolic reprogramming, or excessive mitochondrial fission (Cheng and Mruk, 2012; Feng et al., 2022; Haas et al., 2022; Kabbesh et al., 2022; Zhao et al., 2022). Mitochondrial injury may participate in these events, but its position in the causal sequence differs among models. In arsenic-exposed models, mitochondrial ROS-associated stress signaling was linked to blood–testis barrier disruption, and melatonin exposure was associated with preservation of barrier-related endpoints (Niu et al., 2025). In lipopolysaccharide-exposed calf Sertoli cells, melatonin reduced Toll-like receptor 4 (TLR4)/myeloid differentiation primary response 88 (MyD88)/NF-κB-associated signaling and junctional injury (Feng et al., 2022), whereas heat-stressed sheep Sertoli cells showed melatonin-associated metabolic adaptation involving heat shock protein 90 (HSP90)/hypoxia-inducible factor 1α (HIF-1α)-related glucose metabolism (Deng et al., 2022). Together, these models support context-dependent regulation of inflammatory, metabolic, and junctional responses by melatonin, but they do not establish direct functional repair of the human blood–testis barrier (**Figure 1C**). Increased junctional-protein expression should therefore be distinguished from restored barrier selectivity, which requires functional permeability, ultrastructural assessment, germ-cell transit, and reproductive endpoints.

#### 3.1.4 Developing germ cells and stage-specific quality control

Spermatogonia, spermatocytes, and spermatids have distinct metabolic and quality-control requirements and depend on Sertoli-cell metabolites (Boussouar and Benahmed, 2004; Rajender et al., 2010; Vertika et al., 2020; Park and Pang, 2021). Germ cells are vulnerable to oxidative DNA damage and mitochondria-associated apoptosis, yet physiological apoptosis is required to remove abnormal cells (Shaha et al., 2010; Aitken and Curry, 2011; Tait and Green, 2013; Bock and Tait, 2020). Melatonin reduced oxidative apoptosis in a busulfan-exposed mouse spermatogonial stem-cell model (Li et al., 2018; Zhang et al., 2019), promoted haploid-cell development in sheep culture (Deng et al., 2016), and improved cryopreserved testicular tissue in association with ROS/PINK1–Parkin mitophagy (Ma et al., 2026). These findings are model-specific and do not establish restored fertility. Evaluation should include progression of meiosis, production of sperm, integrity of DNA, fertilization, pregnancy and the health of offspring.

#### 3.1.5 Mature spermatozoa: bioenergetics and redox signaling

Mature spermatozoa have midpiece mitochondria involved in oxidative phosphorylation, calcium regulation, redox signaling, and motility (Ruiz-Pesini et al., 2000; Aitken and Curry, 2011; Liu H. et al., 2024; Zhao et al., 2025). Variations in mitochondrial DNA have been linked to human sperm motility, suggesting a role of mitochondrial genotype in sperm function (Ruiz-Pesini et al., 2000). There is, however, a relative difference in mitochondrial oxidative phosphorylation and glycolysis in sperm motility among various species and experimental conditions and different regions of the flagellum. Therefore, stating that sperm motility is solely dependent on mitochondrial ATP is overly simplistic. During capacitation and fertilization-associated signaling, physiological ROS generation occurs, while excessive mitochondrial or non-mitochondrial ROS decreases membrane fluidity and causes axonemal protein degradation, mitochondrial membrane potential impairment, and DNA fragmentation (Baskaran et al., 2021; Bravo et al., 2024; Onochie et al., 2025). The dual role of ROS is especially relevant in sperm because both excessive oxidation and indiscriminate antioxidant supplementation may interfere with normal redox-dependent maturation. Melatonin has been associated with improved sperm mitochondrial function, membrane potential, viability, and motility in several animal and *in vitro* models (He et al., 2016; Xu D. et al., 2020; Wang C. et al., 2025; Wen et al., 2025). In rabbit spermatozoa, MT1/PKC-associated signaling was implicated in the regulation of motility and kinematics (Wang C. et al., 2025). In human spermatozoa, melatonin reduced apoptosis-associated changes through receptor- and ERK-dependent mechanisms (Espino et al., 2011) and limited oxidative stress-induced DNA damage ex vivo (Bejarano et al., 2014). These findings do not establish that oral or systemic melatonin improves natural conception or live birth. Direct semen supplementation and systemic treatment therefore remain pharmacokinetically and biologically distinct interventions (**Figure 1D**). The major cell-specific mitochondrial roles, reported melatonin responses, and principal evidence limitations are summarized in **Table 1**.

**Table 1:** Cell-specific mitochondrial roles and melatonin-responsive evidence in male reproduction.

Cell type	Mitochondrial role	Reported melatonin-associated effect	Evidence and limitations	References
Leydig cells	Steroidogenesis, redox balance, and survival	Preserved steroidogenic proteins and reduced injury	Animal/cell evidence; effects vary by species and context	(Payne and Hales, 2004; Miller and Auchus, 2011; Zirkin and Papadopoulos, 2018; Gao et al., 2019; Ma et al., 2021; Yang et al., 2021; Zhang et al., 2022; Zhu et al., 2022; Ma et al., 2023a; Pan et al., 2023; Chen et al., 2024; Liu et al., 2025)
Sertoli cells	Metabolic support, dynamics, and BTB maintenance	Reduced inflammatory and junctional injury; improved heat adaptation	Livestock/toxicant models; human BTB function unproven	(Cheng and Mruk, 2012; Alves et al., 2013; Oliveira et al., 2015; Deng et al., 2022; Feng et al., 2022; Haas et al., 2022; Kabbesh et al., 2022; Zhao et al., 2022; Niu et al., 2025)
Germ cells	Stage-specific metabolism, mitophagy, and apoptosis	Reduced oxidative injury; improved development or tissue preservation	Model-specific; fertility and offspring outcomes are rarely tested	(Boussouar and Benahmed, 2004; Rajender et al., 2010; Shaha et al., 2010; Tait and Green, 2013; Deng et al., 2016; Zhang et al., 2019; Vertika et al., 2020; Park and Pang, 2021; Ma et al., 2026)
Spermatozoa	OXPHOS, calcium handling, and redox signaling	Improved motility, viability, membrane potential, or DNA integrity	Animal and ex vivo human evidence; no proven live-birth benefit	(Ruiz-Pesini et al., 2000; Espino et al., 2011; Amaral et al., 2013; Bejarano et al., 2014; Xu D. et al., 2020; Baskaran et al., 2021; Abruzzese et al., 2024; Liu H. et al., 2024; Onochie et al., 2025; Wang C. et al., 2025; Wen et al., 2025; Meng et al., 2026; Rong et al., 2026)
Immune/stromal cells	Mitochondrial danger signaling and inflammatory amplification	Reduced whole-testis inflammatory markers	Mostly indirect or mixed-tissue evidence	(Bours et al., 2006; West et al., 2011; Iyer et al., 2013; Weinberg et al., 2015; Salehi et al., 2019; Samanta, 2020; Kumar et al., 2021a; Feng et al., 2022; Niu et al., 2024)

### 3.2 Melatonin availability as a determinant of redox modulation

#### 3.2.1 Pineal and extrapineal sources

Circulating melatonin is taken up and distributed. It can then be modified, interact with receptors, or be destroyed. Local synthesis of melatonin from the amino acid tryptophan can occur in reproductive tissue. The pineal gland releases melatonin at night using the enzymes tryptophan hydroxylase, aromatic amino-acid decarboxylase, arylalkylamine N-acetyltransferase (AANAT) and acetylserotonin O-methyltransferase (ASMT) (Gonzalez-Arto et al., 2016; Coelho et al., 2019; Rios et al., 2023; Pundkar et al., 2025)(**Figure 2A**). Circulating melatonin reaches reproductive tissues, but responses vary with species and season. Pinealectomy alters testicular receptor, synthetic-enzyme, and RORα rhythms (Baburski et al., 2015; Coelho et al., 2019), yet it also disrupts broader neuroendocrine and circadian organization, so its effects cannot be attributed solely to direct testicular mitochondrial signaling.

**Figure 2:**
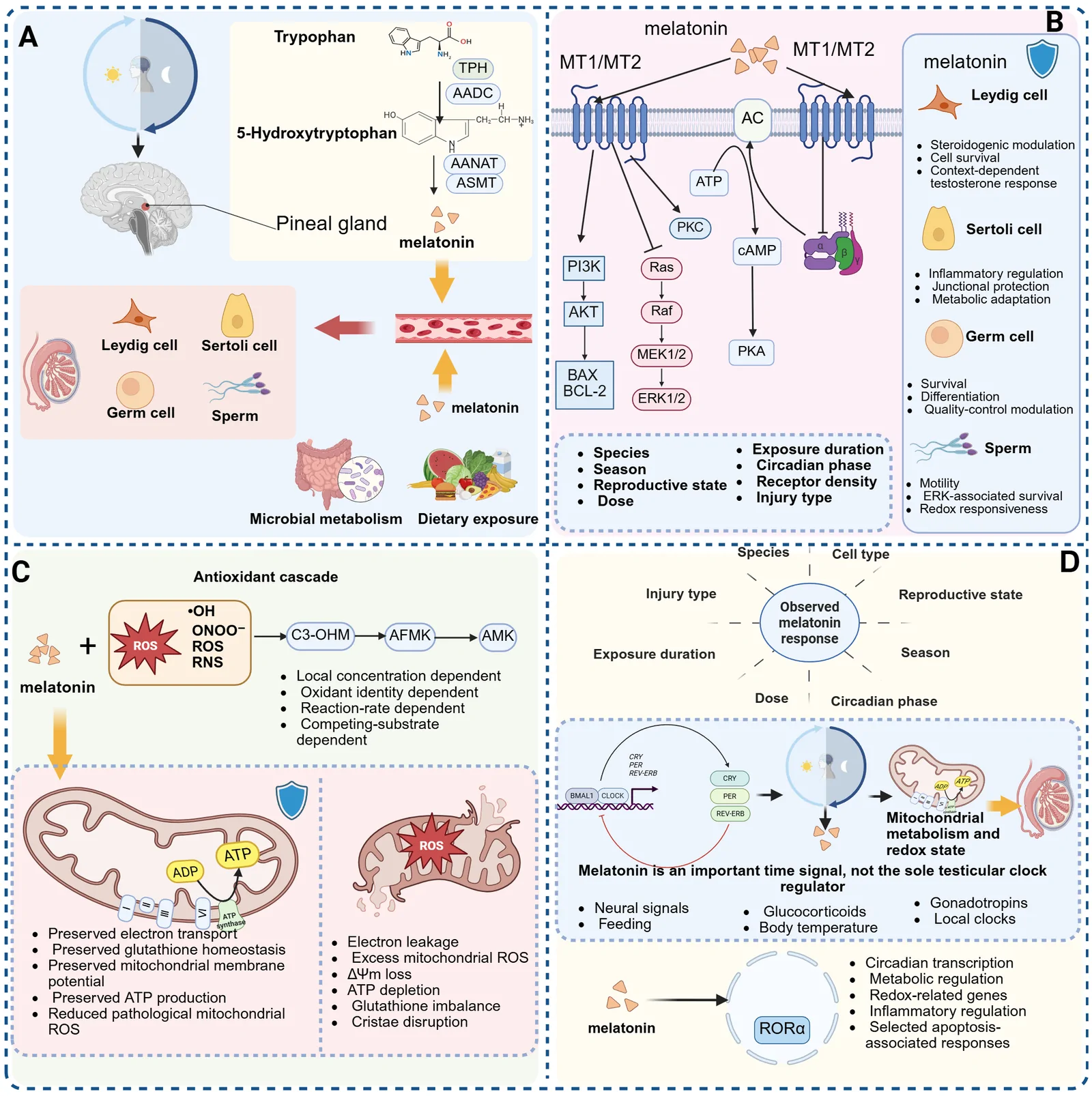
Melatonin availability, signaling, and mitochondria-associated actions in male reproductive tissues. **(A)** Systemic sources, potential exposure modifiers, and tissue distribution. **(B)** Context-dependent MT1/MT2-associated signaling. **(C)** Chemical redox and mitochondria-associated actions. **(D)** Circadian and experimental determinants of melatonin responses. RORα is presented as a possible downstream or parallel regulator rather than a confirmed melatonin receptor.

Melatonin and synthetic enzymes have been reported in extrapineal and male reproductive tissues, including the ram reproductive tract (Acuña-Castroviejo et al., 2014; Gonzalez-Arto et al., 2016). Local production may support autocrine or paracrine regulation, but enzyme expression does not establish synthesis rate, cellular origin, or physiological significance. Human evidence is limited to observational findings in infertile testicular samples (Rossi et al., 2014), which do not establish treatment efficacy. Mitochondrial detection and accumulation are supported (Jou et al., 2007; Tan et al., 2007; Venegas et al., 2012; Reiter et al., 2017; Reiter et al., 2020; Tan et al., 2023), but may reflect uptake, partitioning, binding, transport, local synthesis, or combinations of these processes. Accumulation therefore cannot be used as proof of autonomous synthesis.

#### 3.2.2 Mitochondrial accumulation versus putative synthesis

The hypothesis that mitochondria possess an evolutionarily conserved capacity to synthesize melatonin was proposed on the basis of their endosymbiotic origin and the distribution of melatonin-synthetic pathways (Tan et al., 2013). Subsequent studies reported mitochondrial melatonin synthesis in mouse and porcine oocytes (He et al., 2016; Zhu et al., 2024), while Suofu et al. identified melatonin-synthetic machinery and associated signaling in neuronal mitochondria (Suofu et al., 2017). These studies provide proof-of-concept evidence but do not establish autonomous melatonin synthesis in male reproductive mitochondria. The available direct evidence derives from neuronal systems and female gametes (He et al., 2016; Suofu et al., 2017; Zhu et al., 2024); therefore, mitochondrial melatonin synthesis in male reproductive tissues remains a hypothesis. No cited study directly demonstrates *de novo* synthesis in purified human Leydig-cell, Sertoli-cell, germ-cell, or sperm mitochondria. Evidence from neuronal mitochondria and mammalian oocytes therefore suggests that some mitochondria may contain melatonin-synthetic machinery, whereas functional *de novo* synthesis in male reproductive mitochondria remains unresolved. Rigorous confirmation would involve contamination-controlled organelle purification, isotope tracing, measuring enzymatic activity, manipulation of AANAT or ASMT genetically, and verification in human reproductive samples. Diffusion and membrane partitioning are some other possible explanations in the context of the cellular uptake of melatonin because melatonin has amphiphilic properties (Reiter et al., 2016). Though diffusion may explain some of the phenomena, it may not fully explain the accumulation in tissues and organelles, cellular concentration gradients, and rapid cellular uptake.

#### 3.2.3 Cellular uptake and emerging noncanonical sources

Data from non-reproductive systems suggest some members of the glucose-transporter and proton-coupled oligopeptide-transporter families, including GLUT/SLC2 carriers and PEPT1/PEPT2, may help transport melatonin to cells and mitochondria (Hevia et al., 2015; Huo et al., 2017). From the perspective of transport biology, this is still considered novel. It is currently unclear if any of these or other transporters control the uptake of melatonin by human Leydig cells, Sertoli cells, germ cells, or sperm. It is not known whether melatonin is imported to the mitochondria, and it should be considered that the same machinery could be involved in melatonin uptake by the plasma membrane. Just the presence of a transporter does not indicate that melatonin is a substrate of physiological importance. To resolve the mechanism by which melatonin is imported to the mitochondria and retained in the cell, it will be necessary to conduct melatonin uptake studies while perturbing the various carriers and using organelle-specific analysis. Melatonin can also be found in plants and animals, and it has been proposed that diet alters melatonin or metabolite concentrations in the circulation (Salehi et al., 2019; Zanirate et al., 2026). Foods that contain melatonin also contain polyphenols, vitamins, minerals, lipids, and many other active substances (Zanirate et al., 2026). For this reason, the association of these foods with the different parameters of reproduction is not sufficient to evaluate the role of melatonin in these processes, since circulating melatonin is rapidly metabolized and may vary in a circadian fashion.

In the study, the authors give early evidence that the gastrointestinal sources may be relevant to sheep as a ruminant species regarding the relationship between melatonin sources in the rumen and reproductive physiology (Niu et al., 2024). This model does not establish that dietary melatonin contributes meaningfully to serum or testicular melatonin in humans, and current evidence does not support melatonin-rich foods as a specific treatment for male infertility. The gut microbiota can influence male reproductive physiology through metabolic, immune, endocrine, and redox pathways. High-fat-diet-associated gut dysbiosis has been linked to testicular inflammation and ferroptosis in experimental models (Zhang Z. et al., 2024), supporting a gut–testis axis without demonstrating mediation by microbiota-derived melatonin. Some microorganisms may synthesize or metabolize indole compounds, including melatonin or related precursors (Konopelski and Ufnal, 2018; Que et al., 2020). The quantitative contribution of microbial melatonin to circulating or testicular pools remains unknown, as does the distinction between *de novo* microbial production, medium-derived melatonin, and host–microbial co-metabolism. Microbiota-derived melatonin should therefore be regarded as an unresolved source whose physiological and reproductive relevance requires direct quantitative validation (**Figure 2A**).

Intercellular transfer of mitochondria has been reported in several injury models through proposed routes that include tunneling nanotubes, extracellular vesicles, cell fusion, and release or uptake of free mitochondria (Iorio et al., 2024). Stem or stromal cells may donate mitochondria to injured recipient cells and thereby influence bioenergetics, calcium handling, or survival (Iorio et al., 2024). Evidence from non-reproductive tissues further suggests that melatonin may promote mitochondrial transfer or improve the viability and function of transferred mitochondria (Nasoni et al., 2021). This concept is potentially relevant to testicular injury because Sertoli cells, Leydig cells, germ cells, peritubular cells, macrophages, and stem-cell populations communicate closely. However, no direct evidence in the cited literature shows that melatonin promotes mitochondrial donation to damaged male reproductive cells or that such transfer restores spermatogenesis. The existence, direction, frequency, and physiological significance of mitochondrial transfer within the seminiferous epithelium therefore remain unresolved. Taken together, pineal secretion is the established source of circulating melatonin, whereas local reproductive synthesis is supported mainly by animal evidence and remains poorly quantified in humans (He et al., 2016; Suofu et al., 2017; Zhu et al., 2024). While the mechanism of mitochondrial accumulation is better understood than that of autonomous synthesis of melatonin, transporter-mediated uptake, dietary or microbial contributions, and intercellular mitochondrial transfer remain either emerging or hypothetical. These mechanisms should be considered as potential effect modifiers of melatonin and repair of tissues until direct evidence of reproduction is available (Jou et al., 2007). Current evidence for systemic, local, mitochondrial, transporter-mediated, dietary, microbial, and intercellular determinants of melatonin availability is summarized in **Table 2**.

**Table 2:** Evidence for melatonin sources, uptake, and intracellular availability.

Source/process	Evidence status	Male reproductive evidence	Interpretation	References
Pineal secretion	Established	Animal studies link systemic rhythms with testicular physiology	Established systemic source; reproductive effects remain context dependent	(Reiter, 1991; Gonzalez-Arto et al., 2016; Coelho et al., 2019)
Local reproductive synthesis	Supported in selected tissues	Strongest in the ram reproductive tract; limited human observations	Cellular contribution in humans remains uncertain	(Reiter, 1991; Acuña-Castroviejo et al., 2014; Rossi et al., 2014; Gonzalez-Arto et al., 2016; Perumal et al., 2020; Hassan et al., 2022; Jauhari et al., 2025)
Mitochondrial accumulation	Supported	No quantitative proof in purified human reproductive mitochondria	Better supported than autonomous synthesis	(Jou et al., 2007; Tan et al., 2007; Venegas et al., 2012; Reiter et al., 2017; Reiter et al., 2020; Tan et al., 2023)
Mitochondrial synthesis	Hypothetical in male tissues	Reported in neuronal mitochondria and oocytes only	Requires direct isotope-tracing evidence	(Tan et al., 2013; He et al., 2016; Suofu et al., 2017; Zhu et al., 2024)
Passive diffusion	Plausible	Not quantified in reproductive cells	Likely contributes to uptake	(Reiter et al., 2016)
GLUT/PEPT transport	Emerging	Not established in male reproductive cells	Requires transporter-specific validation	(Hevia et al., 2015; Huo et al., 2017)
Dietary or microbial sources	Emerging	No causal male-fertility evidence	Potential exposure modifiers, not therapies	(Konopelski and Ufnal, 2018; Salehi et al., 2019; Que et al., 2020; Niu et al., 2024; Zhang Z. et al., 2024; Zanirate et al., 2026)
Mitochondrial transfer	Hypothetical in the testis	Shown only in non-reproductive injury models	Future research direction	(Nasoni et al., 2021; Iorio et al., 2024)

### 3.3 Modulating ROS-associated reproductive injury

#### 3.3.1 MT1 and MT2 as established membrane receptors

Melatonin-responsive effects may involve established MT1/MT2 signaling, circadian organization, chemical interactions with reactive species, and mitochondria-associated processes. MT1 and MT2 are established pharmacological melatonin receptors (Dubocovich et al., 2010), whereas direct physiological binding of melatonin to RORα remains controversial. Mitochondrial accumulation of melatonin is supported (Venegas et al., 2012; Huo et al., 2017; Reiter et al., 2017; Ma et al., 2026), but receptor independence is rarely demonstrated in reproductive models. Associations of melatonin exposure with altered AKT, extracellular signal-regulated kinase (ERK), nuclear factor erythroid 2-related factor 2 (NRF2), SIRT1, ROS, or mitochondrial membrane potential are therefore interpreted as evidence of pathway engagement rather than pathway causality when based only on expression, phosphorylation, or biochemical markers. Stronger causal inference requires direct perturbation of the proposed receptor or signaling node, compatible temporal ordering, and, where feasible, rescue of the relevant cellular or reproductive phenotype (Yao et al., 2019; Chumboatong et al., 2020; Zhang et al., 2022; Habiba et al., 2023; Chen et al., 2024; Zhao et al., 2024; Wang C. et al., 2025). MT1 and MT2, encoded by MTNR1A and MTNR1B, show species-, season-, stage-, and cell-specific expression (Rossi et al., 2014; Gao et al., 2019; Zhang et al., 2022). Receptor depletion altered human chorionic gonadotropin-stimulated testosterone secretion in mouse Leydig cells (Gao et al., 2019), and MT1/protein kinase C signaling was implicated in rabbit sperm motility (Wang C. et al., 2025). Human sperm ex vivo also showed receptor/ERK-associated protection (Espino et al., 2011). Receptor expression alone, however, is insufficient to assign an outcome to a specific receptor subtype.

#### 3.3.2 G-Protein coupling and kinase crosstalk

MT1/MT2 responses depend on receptor density, homo- or heteromerization, G-protein coupling, ligand concentration, exposure duration, desensitization, intracellular localization, and concurrent hormonal signals (Dubocovich et al., 2010). Most evidence derives from animal models (Gerits et al., 2008; Xu et al., 2021; Jin et al., 2022; Zhu et al., 2022), whereas human data remain limited to testicular observations and isolated sperm studies (Espino et al., 2011; Bejarano et al., 2014; Rossi et al., 2014; Riviere et al., 2020).

Both receptors commonly couple to Gi/o and can reduce adenylyl cyclase activity (Dubocovich et al., 2010). This complicates a simple MT1/MT2–cAMP/PKA–StAR model because LH/hCG stimulates Leydig-cell steroidogenesis through cAMP/PKA (Gerits et al., 2008; Xu et al., 2021; Jin et al., 2022; Zhu et al., 2022). Reported effects may instead involve alternative coupling, β-arrestin or kinase signaling, indirect control of StAR through PI3K/AKT or ERK, circadian regulation, or preservation of the steroidogenic machinery during injury (Jia et al., 2018; Sun et al., 2022; Ma et al., 2023b). Direction and magnitude are therefore context-dependent (**Figure 2B**).

#### 3.3.3 Cell-specific receptor responses

In Leydig cells, receptor depletion or melatonin supplementation alters steroidogenic responses in mammalian models (Gao et al., 2019; Yang et al., 2021), whereas melatonin exposure was associated with reduced testosterone synthesis and altered lipid-droplet lipolysis in rooster Leydig cells (Zhu et al., 2022). These opposing observations argue against a universal stimulatory effect and instead support context dependence according to species, dose, circadian phase, reproductive state, and metabolic condition. PI3K/AKT activation has been associated with protection in toxicant-exposed swine testicular cells and mouse testes (Sun et al., 2022; Chen et al., 2024), whereas an MT2/AKT-associated response accompanied reduced mitochondrial dysfunction and ferroptosis-related injury in swine testicular cells (Zhao et al., 2024). These findings support involvement of PI3K/AKT signaling in selected models but do not establish a universal linear MT1/MT2→AKT pathway in the absence of receptor-specific loss-of-function, temporal ordering, and rescue evidence. Similarly, receptor/ERK-associated protection in human sperm (Espino et al., 2011), MT1/protein kinase C-associated regulation of rabbit sperm motility (Wang C. et al., 2025), and p38 mitogen-activated protein kinase-associated inflammatory responses in ovine orchitis (Deng et al., 2020) represent distinct experimental contexts rather than components of one conserved melatonin signaling cascade. Representative pathways and their principal evidence boundaries are summarized in **Table 3**.

**Table 3:** Melatonin-responsive signaling in male reproductive models.

Pathway	Representative model/cell	Reported effect	Evidence boundary	References
MT1/MT2	Mouse Leydig cells	Altered hCG-stimulated testosterone production	Direct reproductive evidence; receptor-subtype roles remain unclear	(Dubocovich et al., 2010; Gao et al., 2019)
MT1/PKC	Rabbit sperm	Regulated motility and kinematics	Direct but species-specific ex vivo evidence	(Wang C. et al., 2025)
Receptor/ERK	Human sperm	Reduced apoptosis-associated changes	Ex vivo evidence; no fertility endpoint	(Espino et al., 2011)
SIRT1/NRF2	Cisplatin-exposed mouse testis/Leydig cells	Reduced oxidative injury	Toxicant-specific; pathway order unresolved	(Zhang et al., 2022)
PI3K/AKT	TBBPA/DEHP testicular models	Reduced apoptosis and mitochondrial injury	Receptor dependence not uniformly demonstrated	(Sun et al., 2022; Chen et al., 2024)
MT2/AKT	Swine testicular cells	Reduced ferroptosis and mitochondrial dysfunction	Non-human model; downstream mechanism incomplete	(Zhao et al., 2024)
TLR4/MyD88/NF-κB	LPS-exposed calf Sertoli cells	Reduced inflammation and junctional injury	Livestock neonatal model	(Feng et al., 2022)
HSP90/HIF-1α	Heat-stressed sheep Sertoli cells	Improved metabolic adaptation	Heat- and species-specific	(Deng et al., 2022)
p38 MAPK	Ovine orchitis	Reduced inflammation and oxidative stress	Mixed-cell tissue response	(Deng et al., 2020)
RORα-associated signaling	Rodent Leydig/testis models	Apoptosis or circadian associations	RORα is not a confirmed melatonin receptor	(Coelho et al., 2019; Li et al., 2020)
Chemical/mitochondrial actions	Biochemical and injury models	Reactive-species neutralization and bioenergetic protection	Direct receptor-independent testicular action remains unproven	(Martín et al., 2000; Martín et al., 2002; Reiter et al., 2003; Jou et al., 2007; Tan et al., 2007; Hardeland et al., 2009; Venegas et al., 2012; Reiter et al., 2017)

#### 3.3.4 Circadian coordination of testicular signaling

Melatonin correlates with environmental darkness and synchronizes the central and peripheral circadian systems (Reiter, 1991; Partch et al., 2014; Claustrat and Leston, 2015; Tordjman et al., 2017). Circadian rhythms also exist in the male reproductive system. There are circadian rhythms of testosterone in the blood and sleep disturbance has been associated with reduced semen quality (Bremner et al., 1983; Jensen et al., 2013). Testicular clock genes and Leydig cell endocrine activity show circadian rhythms in research studies (Alvarez et al., 2008; Baburski et al., 2015; Meneses-Santos et al., 2018; Coelho et al., 2019). In the CLOCK–BMAL1 transcriptional complex at the cellular level, feedback circadian systems control metabolism and hormonal production, and modulate redox status and the response to stress (Koike et al., 2012; Partch et al., 2014; Takahashi, 2017). BMAL1 deficiency impacts both fertility and testosterone production and further establishes a relationship with male reproductive physiology (Alvarez et al., 2008). There are circadian rhythms in mitochondrial respiration, NAD^+^ metabolism, substrate metabolism, and cellular redox status (Peek et al., 2013; Putker and O'Neill, 2016; de Goede et al., 2018). These observations suggest a bidirectional relationship: circadian clocks regulate mitochondrial metabolism, while redox and metabolic state can feed back on clock function (**Figure 2D**). However, melatonin should not be described as the sole coordinator of testicular circadian physiology. Neural signals, feeding cycles, glucocorticoids, body temperature, gonadotropins, and local clocks may also entrain reproductive tissues. Furthermore, associations between sleep disturbance and semen quality (Jensen et al., 2013) do not prove that reduced melatonin mediates the relationship. The evidence therefore separates established circadian functions of systemic melatonin, rhythmic testicular and Leydig-cell physiology, associations between sleep and human semen quality, experimental links between clock genes and fertility, and the unresolved contribution of local melatonin signaling.

#### 3.3.5 RORα-associated signaling and receptor controversy

RORα is a nuclear receptor and circadian transcriptional regulator involved in metabolic, inflammatory, and redox-related gene expression. Its testicular expression is rhythmic in rats, and pinealectomy altered the phase of that rhythm; melatonin replacement partially restored selected changes (Coelho et al., 2019). In mouse Leydig cells, RORα/p53 signaling was associated with melatonin-mediated suppression of apoptosis (Li et al., 2020). These observations support the participation of RORα in selected melatonin-responsive processes, but they do not establish direct ligand–receptor binding. RORα overexpression also protected neurons from oxidative-stress-induced apoptosis (Boukhtouche et al., 2006), which provides only indirect, non-reproductive evidence. RORα should therefore be interpreted as a potential downstream or parallel transcriptional regulator rather than a confirmed melatonin receptor (**Figure 2D**). Changes in RORα after melatonin exposure could reflect altered circadian phase, redox state, kinase signaling, metabolic cofactors, or transcriptional feedback. Direct binding at physiological concentrations, RORα-selective genetic models, chromatin-occupancy studies, and separation from MT1/MT2 signaling are needed to resolve this mechanism.

#### 3.3.6 Receptor-independent and receptor-nonexclusive actions

Melatonin and its metabolites can react chemically with reactive oxygen and nitrogen species, forming an antioxidant cascade that does not require membrane-receptor activation (Reiter et al., 2003; Tan et al., 2007; Hardeland et al., 2009). Because of its amphiphilicity, it can enter cells. Isolated or non-reproductive mitochondrial preparations show associations with preserved electron transport, glutathione, membrane potential, and ATP (Martín et al., 2000; Martín et al., 2002; Jou et al., 2007; Venegas et al., 2012; Reiter et al., 2016; Reiter et al., 2017).

These data support a theory and do not directly test for a biological function of the testes. Mitochondrial accumulation does not prove receptor independence, as MT1/MT2 signaling or the putative intracellular receptor complexes may indirectly influence mitochondria (Suofu et al., 2017). Protection could be the result of reduced inflammation or improved metabolism. Receptor independence must be demonstrated after dual MT1/MT2 deletion and tested in purified mitochondria. The male reproductive studies suggest a protective role of mitochondria, but do not provide a consistent initiating pathway. Therefore, “receptor-nonexclusive mitochondria-associated action” is the more reasonable conclusion (**Figure 2C**).

## 4 Mitochondrial integration of redox, inflammatory, and cell-death stress responses

Oxidative stress, mitochondrial dysfunction, inflammatory signaling, and regulated cell death frequently coexist in experimental models of male reproductive injury, but their temporal order and causal relationships vary among initiating insults, cell types, species, and experimental systems. The following sections therefore examine redox and mitochondrial quality-control responses, mitochondrial–inflammatory crosstalk, and regulated cell-death-associated injury separately before integrating these observations into a proposed interpretive framework.

### 4.1 Redox imbalance and mitochondrial quality-control failure

#### 4.1.1 Physiological and pathological mitochondrial ROS

Mitochondrial electron transport is one source of intracellular ROS through electron leakage and partial reduction of molecular oxygen (Murphy, 2009). As discussed in Section 3.1.1, low and transient ROS can support metabolic adaptation, calcium signaling, steroidogenesis, and fertilization-related sperm responses (Brookes et al., 2004; Murphy, 2009; Baskaran et al., 2021). Excessive ROS can lead to pathology when repair and antioxidant mechanisms become overextended. Under these conditions, the effects of ROS can extend to the mitochondria and include the oxidation of proteins of the respiratory chain and of phospholipids of the membrane, and the disruption of mitochondrial membrane potential, calcium handling, the production of ATP, and the integrity of mtDNA (Murphy, 2009; Galluzzi et al., 2012; Nunnari and Suomalainen, 2012; Chandel, 2015; Bock and Tait, 2020). The damage caused to respiratory-chain components can eventually lead to an increased leakage of electrons and create a feed-forward loop. Although ROS are necessary for sperm capacitation and acrosomal responsiveness, the protective effects of melatonin may be evaluated as the opening of a redox window rather than as the complete elimination of ROS.

Mitochondrial injury happens in the toxicant, particulate, heat, and metabolic models. There are also NADPH oxidases, inflammatory leukocytes, peroxisomes, endoplasmic reticulum reactions, and exogenous oxidants causing mitochondrial injury (**Figure 3A**) (Morgan and Liu, 2011; Zhao et al., 2024). The effects vary for Leydig cells, Sertoli cells, germ cells, and sperm. These findings indicate mitochondrial oxidative stress is an important aspect of some disorders, but is not considered the only factor or the initial contributor.

**Figure 3:**
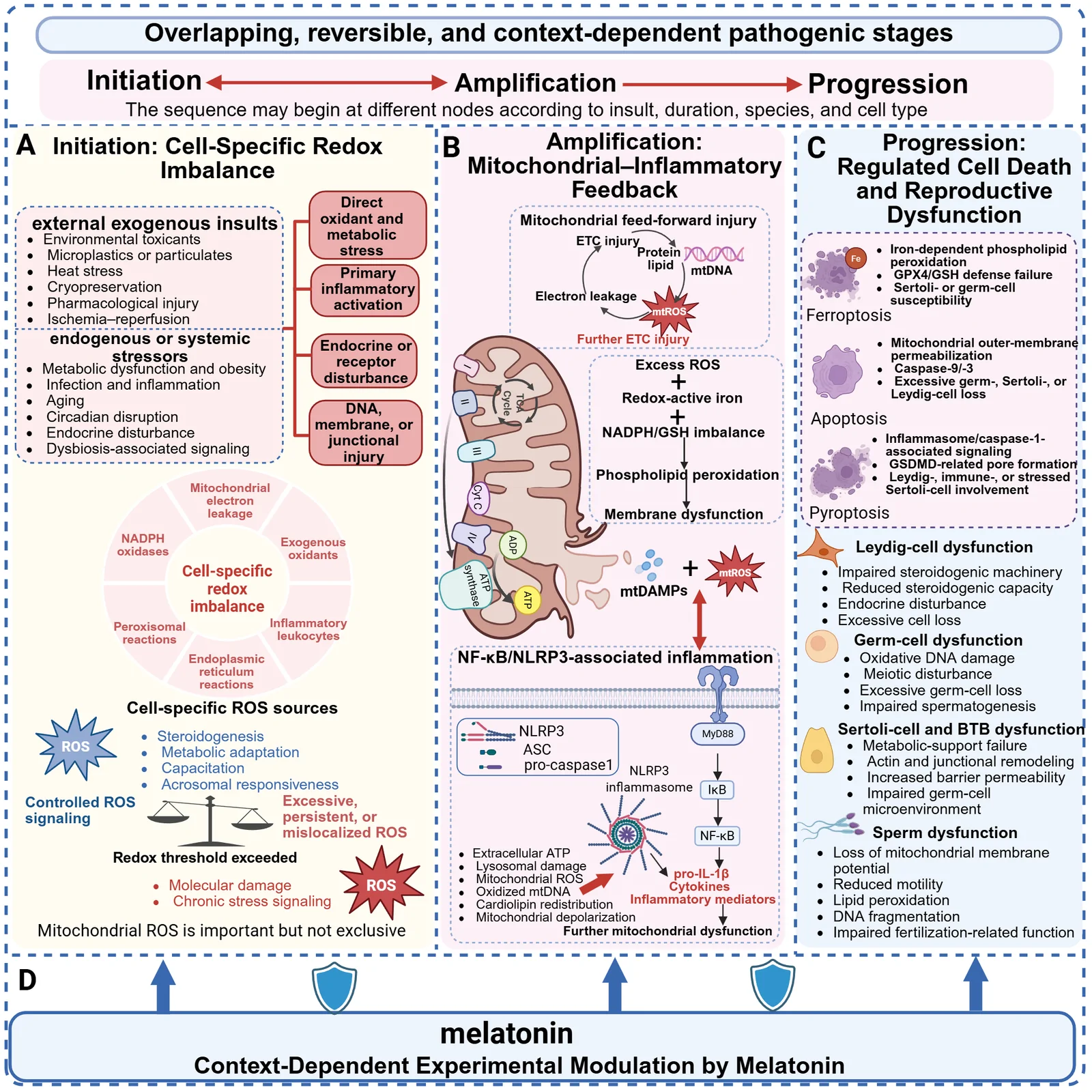
Proposed stage-based synthesis of ROS-associated male reproductive injury and melatonin-responsive modulation. The framework organizes the reviewed evidence into overlapping and context-dependent pathogenic stages. **(A)** Initiation: cell-specific redox imbalance induced by exogenous, endogenous, and systemic stressors, distinguishing physiological ROS signaling from excessive or persistent oxidative stress. **(B)** Amplification: mitochondrial–inflammatory feedback involving mitochondrial ROS generation, oxidative damage, and NF-κB/NLRP3-associated inflammatory signaling. **(C)** Progression: regulated cell-death-related pathways, including ferroptosis, apoptosis, and pyroptosis, together with Leydig-cell, germ-cell, Sertoli-cell, blood–testis barrier, and sperm dysfunction. **(D)** Context-dependent experimental modulation by melatonin across the proposed stages. These stages may overlap or occur in different sequences, and mitochondrial dysfunction may be primary or secondary depending on the experimental model. Melatonin is presented as a context-dependent modifier rather than a universal upstream inhibitor.

#### 4.1.2 Chemical antioxidant actions and bioenergetic protection

Melatonin engages with several reactive oxygen and nitrogen species and can chemically convert hydroxyl-radical- and peroxynitrite-related species (Reiter et al., 2003; Tan et al., 2007; Galano et al., 2011). The resulting metabolites, including cyclic 3-hydroxymelatonin, N¹-acetyl-N²-formyl-5-methoxykynuramine, and N¹-acetyl-5-methoxykynuramine, demonstrate redox activity and may contribute to an antioxidant cascade (Tan et al., 2007; Hardeland et al., 2009). Most of their physiological functions are dependent on the local concentration of melatonin, the identity and rate of oxidation, intracellular distribution, and the presence of competing substrates. The majority of reproductive studies use pharmacological doses, without measuring melatonin in plasma, testicular tissue, or mitochondria. In isolated mitochondria or other non-reproductive tissues, melatonin has been shown to preserve respiratory activity, glutathione homeostasis, membrane potential, and ATP synthesis (Martín et al., 2000; Martín et al., 2002; Jou et al., 2007). Similar results have been reported in the reproductive models (Zhao et al., 2024; Liu et al., 2025; Niu et al., 2025; Wen et al., 2025), showing that melatonin exposure restores mitochondrial membrane potential and possesses protective effects against cellular injury. Monobutyl phthalate-exposed TM3 Leydig cells (Liu et al., 2025), ferroptosis-prone swine testicular cells (Zhao et al., 2024), and arsenic-exposed testes (Niu et al., 2025) support specific protective effects in the models. However, no evidence is available to support the direct activation of all respiratory-chain complexes in all male reproductive cells. High-resolution respirometry, respiratory-complex activity, coupling efficiency, and intracellular melatonin measurements coupled with receptor-loss studies would be informative to document direct bioenergetic effects.

#### 4.1.3 Sirtuin-, PGC-1α-, FOXO1-, and NRF2-associated adaptation

SIRT1 is an NAD^+^-dependent deacetylase that regulates the adaptation of metabolism, transcription, mitochondrial biogenesis, and stress resistance. Melatonin treatment of monobutyl phthalate-exposed TM3 Leydig cells induced a protective effect by decreasing oxidative stress and apoptosis and was associated with SIRT1/PGC-1α signaling (Liu et al., 2025). While this is the first evidence in a reproductive cell model, it remains unknown if the activation of SIRT1 occurs prior to the recovery of mitochondria or if it is a secondary response to the restoration of redox. PGC-1α is the primary regulator of the mitochondrial biogenesis and oxidative metabolism, but increased PGC-1α expression should not be presumed to improve mitochondrial quality since it also depends on competent respiration and balanced mitophagic flux. In bisphenol-S induced mouse testicular injury, the response to melatonin was associated with NRF2/HO-1 and SIRT1/FOXO1-associated responses (Kumar et al., 2021b), while energy restriction in sheep Leydig cells was associated with SIRT1/superoxide dismutase 2 (SOD2)-dependent protection (Ma et al., 2021).

These observations derive from different species and insults and do not establish a universal SIRT1–PGC-1α–FOXO1–SOD2 cascade. SIRT3 is a mitochondrial deacetylase that can regulate respiratory and antioxidant proteins. Non-reproductive biochemical studies suggest SIRT3-dependent SOD2 deacetylation (Chen et al., 2011; Xiao et al., 2023; Niu et al., 2025), whereas a particulate-matter model linked melatonin with preserved testicular SIRT3 expression and reduced spermatogenic injury (Liu J. et al., 2024). The responsive cell type and downstream substrates were not identified, and the sheep study (Ma et al., 2021) examined SIRT1/SOD2 rather than SIRT3/SOD2. A causal reproductive SIRT3 mechanism therefore requires cell-specific genetic and biochemical validation.

NRF2 regulates antioxidant and detoxification genes through antioxidant-response elements. Melatonin-associated activation or preservation of NRF2 signaling has been reported in mouse testicular and Leydig cell injury models (Kumar et al., 2021b; Zhang et al., 2022; Habiba et al., 2023). PERK-dependent NRF2/antioxidant-response-element signaling also protected mouse spermatocyte-derived cells from dibutyl-phthalate-induced mitochondrial injury (Zhang et al., 2019), but melatonin was not examined in that study. This finding supports the relevance of NRF2 to spermatocyte oxidative injury rather than a direct melatonin–NRF2 mechanism. NRF2 can increase heme oxygenase-1, NAD(P)H quinone oxidoreductase 1, glutathione-related enzymes, and other antioxidant proteins. Its effects depend on duration, cell type, and downstream gene selection; in ferroptosis, NRF2 may influence glutathione synthesis, iron handling, and quinone reduction, but these consequences should not be assumed to be identical across reproductive cells (de Souza et al., 2026).

#### 4.1.4 Mitochondrial dynamics, mitophagy, and biogenesis

Properties of mitochondrial quality control, including fission, fusion, mitophagy, biogenesis, and lysosomal degradation, are interrelated. Studies showed DRP1-associated fission and defective flux in the heat-stressed goat Sertoli cells (Reiter et al., 2017; Wen et al., 2025); Leydig cells treated with cadmium exhibited fission and defective mitophagy (Yi et al., 2022); and melatonin treatment of cryopreserved testicular tissue resulted in the resolution of mitochondrial damage due to excessive levels of ROS and PINK1–Parkin mitophagy (Ma et al., 2026). These studies suggest that mitophagy is context specific; early mitophagy removes ROS and danger signals and prevents respiratory chain inhibition, while excessive or incomplete mitophagy causes a decrease in the respiratory reserve. Therefore, melatonin should be referred to as a potential regulator of quality-control flux.

The interpretation of mitochondrial quality control also relies on the stage of disease. Early mitophagy may remove damaged mitochondrial segments and inhibit the release of mitochondrial danger signals (Jiang et al., 2022). However, in cases of prolonged exposure, the removal of organelles with insufficient compensatory biogenesis may deplete respiratory reserve and affect the maintenance of the barrier or the support of germ cells. Conversely, increased PINK1, Parkin, LC3, or the extent of mitochondrial fission during a given time point may reflect an adaptive response, excessive organelle turnover, or a block in lysosomal degradation. Static abundance may not distinguish these responses (Hu et al., 2016; Lu et al., 2022; Lan et al., 2025). Molecular assessment may provide different responses, protective or maladaptive, in the initiation or progression of the disease, respectively. In heat-stressed Sertoli cells, cadmium-exposed Leydig cells, and cryopreserved testicular tissue, mitochondrial dynamics and mitophagy were evaluated in different injury contexts and outcomes (Yi et al., 2022; Wen et al., 2025; Ma et al., 2026). These models may be compared by the time-resolved assessment of fission and fusion, autophagosome formation and lysosomal delivery, respiratory capacity, mitochondrial mass, and cell-specific function. Thus, the use of melatonin should be described as normalizing quality-control flux, rather than stimulatory or inhibitory of mitophagy. A causal argument requires genetic manipulation of the relevant machinery, along with the restoration of mitochondrial function, as well as a reproductive endpoint, rather than changes in PINK1, Parkin, LC3, p62, and DRP1 alone.

Across these redox, bioenergetic, sirtuin/NRF2, and mitochondrial-quality-control studies, melatonin is most consistently associated with attenuation of pathological oxidative stress and preservation of mitochondrial function in selected experimental models. However, the responsible mechanisms differ among cell types and insults, and these observations do not establish that mitochondrial rescue is the initiating mechanism of melatonin-associated protection in every reproductive context.

### 4.2 Mitochondrial–inflammatory crosstalk through NF-κB and NLRP3

#### 4.2.1 Bidirectional mitochondria–inflammation crosstalk

Damaged mitochondria can increase inflammation due to ROS and damage-associated signals through the release of mitochondrial DNA, cardiolipin, ATP, and N-formyl peptides (Bours et al., 2006; West et al., 2011; Iyer et al., 2013; Weinberg et al., 2015). On the other hand, Toll-like receptor ligands, infection, cytokines, metabolic endotoxemia, tissue injury, and immune-cell infiltration can initiate inflammation and damage mitochondria, independently (**Figure 3B**). Melatonin can have anti-inflammatory effects at different levels, including protection of mitochondria, inhibition of TLRs, activation of MT1/MT2-dependent kinases, transcriptional regulation, and activation of testicular immune cells; their relative importance will be determined by the model employed (Rossi et al., 2014; Samanta, 2020; Feng et al., 2022).

Resident macrophages, Sertoli cells, Leydig cells, peritubular cells, endothelial cells and infiltrating leukocytes all demonstrate differences in mitochondria, antioxidant capacity and cytokine production and therefore in experimental models of inflammation, pulses of cytokine release and inflammatory signals may reach neighboring cells through extracellular ATP, oxidized lipids, mitochondrial DNA, dysfunctional mitochondria or changes in barrier permeability, regardless of whether the recipient cell sustains mitochondrial dysfunction (Bours et al., 2006; West et al., 2011; Weinberg et al., 2015; Deng et al., 2020; Kumar et al., 2021a; Feng et al., 2022). When working with whole-testis homogenates, this cell to cell signal transduction is camouflaged, and it is therefore difficult to decipher whether the increased NF-κB, NLRP3 and IL-1β or oxidative stress has a cellular origin. The effects of melatonin treatment may also be interpreted in different ways due to the inability to maintain the architecture of the testis. Spatial transcriptomics, multiplex imaging, cell-specific knockouts and organotypic culture are complemented by lineage tracing and cytokine analysis to provide important indications of the site of inflammatory origin and how inflammation spreads. Such studies will also help determine the cell type required for the response to melatonin. Such designs may also help to distinguish cell autonomous protection and melatonin response from interruption of a multicellular amplification loop.

#### 4.2.2 NF-κB signaling and parallel inflammatory regulation

NF-κB regulates several inflammatory and stress-responsive genes. In the absence of signaling, NF-κB dimers remain in the cytoplasm by inhibitor-of-κB proteins. Activation of the inhibitor-of-κB kinase complex results in their degradation and the translocation of NF-κB to the nucleus. ROS modulates this pathway through oxidation sensitive kinases, phosphatases and regulatory proteins (Morgan and Liu, 2011), and the impact is dependent on the concentration, ROS compartment, and cell type. Long-term polystyrene-microplastic exposure was associated with inflammatory and aging-related changes in the testis (Wu et al., 2023). In reproductive models, melatonin decreased testicular inflammation induced by lipopolysaccharide in rats through the SIRT1/NF-κB/cyclooxygenase-2 (COX-2) pathway (Kumar et al., 2021a), inhibited TLR4/MyD88/NF-κB signaling and junction injury in neonatal calf Sertoli cells (Feng et al., 2022), and reduced inflammation associated with ovine orchitis (Deng et al., 2020) through regulation of p38 MAPK. These studies support the anti-inflammatory functions of melatonin, but do not clearly support the inhibition of NF-κB as a consequence of mitochondrial repair. The Sertoli-cell study (Feng et al., 2022) suggests that TLR4/MyD88/NF-κB are regulated in concert, and, thus, in addition to the receptor and redox systems, the mitochondrial and inflammatory systems are active. Injury from hydrogen peroxide, studies of microglia, intervertebral discs and colitis (Moniruzzaman et al., 2018; Chamanara et al., 2019; Yao et al., 2019; Chen et al., 2020), provide only indirect evidence to support the role of male reproductive tissues and should not be considered as evidence.

#### 4.2.3 NLRP3 priming, activation, and mitochondrial danger signals

The NLRP3 inflammasome comprises NLRP3, ASC and procaspase-1. NF-κB related priming increases the expression of NLRP3 and pro-interleukin-1β. NLRP3 inflammasome activation happens due to ionic flux, the presence of extracellular ATP, damage to lysosomes, mitochondrial ROS and oxidized mitochondrial DNA, cardiolipin redistribution, and mitochondrial membrane depolarization (Oduro et al., 2022; Zhang W. et al., 2025). Following assembly, caspase-1 matures interleukin-1β, interleukin-18 and cleaves gasdermin D. It is possible that the major role of mitochondrial stress is in the activation of the inflammasome, while priming is likely provided by Toll-like receptors or inflammatory cytokines. NLRP3 activation may occur in non-reproductive systems and may involve oxidized mitochondrial DNA and cardiolipin (Shimada et al., 2012; Iyer et al., 2013). However, there is a lack of evidence showing direct cardiolipin-dependent assembly in Leydig cells, Sertoli cells, and germ cells.

Reproductive studies show model-dependent engagement of these pathways. NLRP3-associated Leydig-cell pyroptosis has been reported in obesity models (Wang et al., 2023; Wei et al., 2025) and toxicant models (Ruan et al., 2024; Dai et al., 2026), while heat stress activated NLRP3-related responses in boar Sertoli cells (Zhang X. et al., 2025). Additional whole-testis studies linked NLRP3-related injury with combined microcystin and nitrite exposure (Liu H. et al., 2024), di(2-ethylhexyl) phthalate exposure (Hong et al., 2021), other chemical-injury models (Elhessy et al., 2025; Kamel and Hussein, 2026), and ischemia–reperfusion (Almarzouq and Al-Maghrebi, 2023). The trihexyl-phosphate study implicated released mitochondrial DNA and cGAS–STING signaling (Dai et al., 2026), which is mechanistically distinct from NLRP3. Collectively, these findings establish that mitochondrial danger and inflammasome pathways operate in reproductive tissues, but not that an identical initiating signal or cell-death sequence applies to every model.

Melatonin reduced 1,2-dichloroethane-induced testicular pyroptosis through a piRNA/IRF7-associated mechanism (Zhang B. et al., 2024), which should not be recast as a generic mtROS–NLRP3 pathway. Melatonin-associated inhibition of NLRP3 or pyroptosis in intervertebral-disc and fish-intestinal models (Chen et al., 2020; Miao et al., 2022) provides indirect, non-reproductive support only. Direct evidence that melatonin prevents cardiolipin-dependent NLRP3 recruitment or oxidized-mitochondrial DNA-dependent inflammasome assembly in male reproductive cells remains insufficient; these mechanisms are plausible but unconfirmed.

Taken together, melatonin may modify mitochondrial–inflammatory crosstalk at several levels, including inflammatory priming, mitochondrial injury, and inflammasome-associated signaling. Current evidence, however, does not identify a single conserved anti-inflammatory mechanism operating across Leydig cells, Sertoli cells, germ cells, and other testicular cell populations. In particular, reduced NF-κB or NLRP3-related markers should not automatically be interpreted as evidence that mitochondrial repair is the initiating event.

### 4.3 Regulated cell death and loss of reproductive function

#### 4.3.1 Mitochondrial apoptotic signaling

Apoptosis is important for normal spermatogenic quality control, but is pathological when germ, Sertoli, or Leydig cells are lost due to injury (Shaha et al., 2010). Oxidative stress, DNA damage, toxicants, heat, and metabolic disturbance all can lead to an elevation in p53 and BAX/BAK levels, which leads to mitochondrial outer-membrane permeabilization and release of cytochrome c. This leads to apoptosome formation and activation of caspase-9/-3 (Shaha et al., 2010; Tait and Green, 2013; H and B, 2020; Han et al., 2020; Fang et al., 2024; Liu H. et al., 2024; Yuan et al., 2024). The expression of these markers in tissue does not specify the affected cell type or infer the potential for restored fertility.

Melatonin attenuated apoptosis-related changes in reproductive models (Espino et al., 2011; Li et al., 2018; Li et al., 2020; Sun et al., 2022; Chen et al., 2024; Liu et al., 2025; Niu et al., 2025). Mechanisms and evidence strength differ (**Figure 4C**). RORα/p53 and SIRT1/PGC-1α derive from mouse Leydig cells (Li et al., 2020) and TM3 cells (Liu et al., 2025), respectively. Melatonin protection in the busulfan model is observed in mouse spermatogonial stem cells (Li et al., 2018). Exposure of human sperm to these factors led to activation of receptors and ERK (Espino et al., 2011). A study using selenium co-treatment (Baş and Nazıroğlu, 2019) did not discriminate between selenium and melatonin. To validate the functions, recovery of germ cells, hormone secretion, sperm DNA integrity, fertilization, pregnancy, and live birth of offspring are needed.

**Figure 4:**
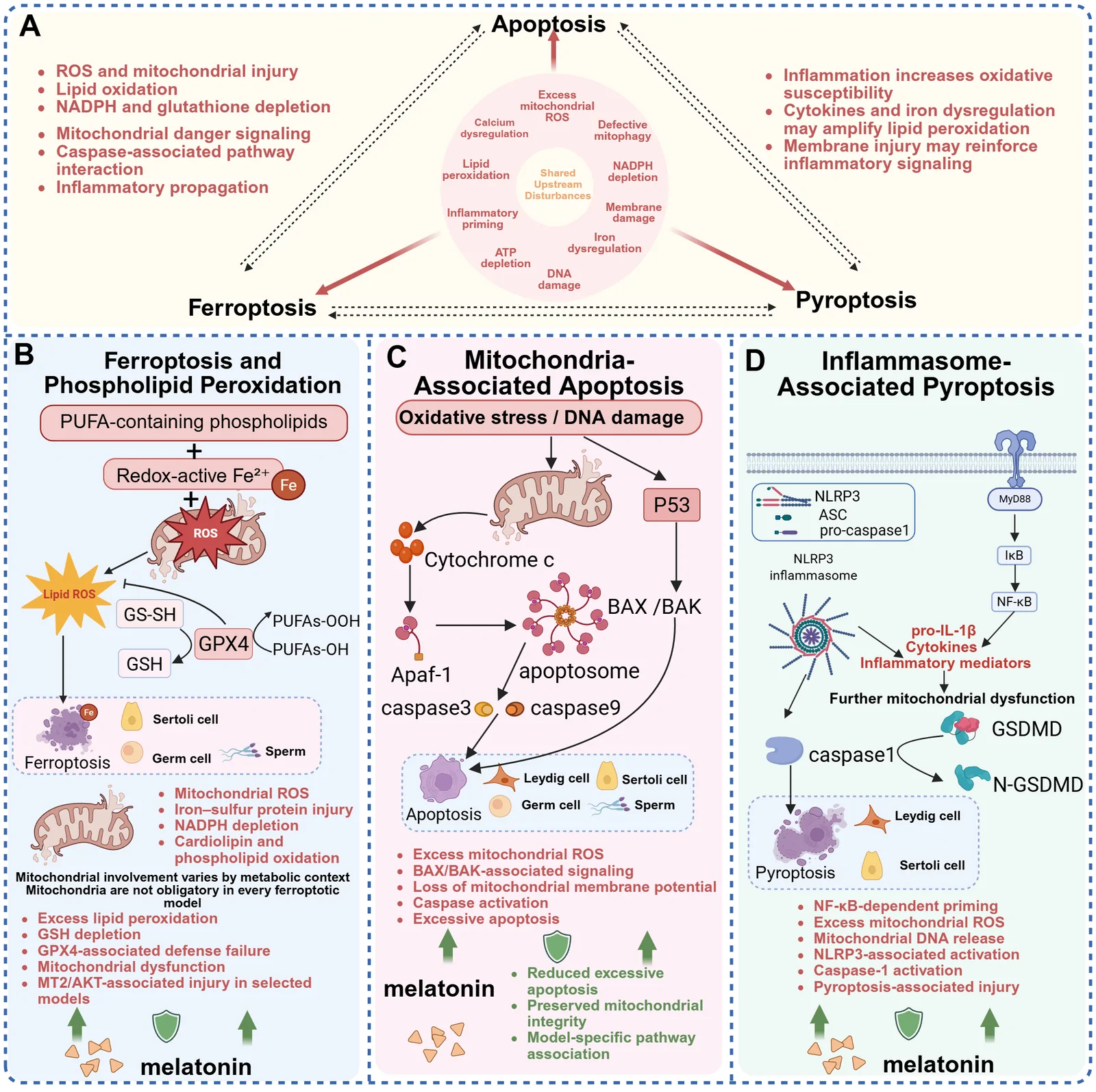
Crosstalk among apoptosis-, ferroptosis-, and pyroptosis-related pathways in male reproductive injury. **(A)** Shared and distinct stress-related features of apoptosis, ferroptosis, and pyroptosis, including mitochondrial injury, oxidative stress, lipid peroxidation, and inflammatory signaling. **(B)** Ferroptosis-associated phospholipid peroxidation involving redox-active iron, glutathione depletion, GPX4-related antioxidant defenses, and mitochondrial injury. **(C)** Mitochondria-associated apoptosis involving oxidative stress, DNA damage, mitochondrial membrane dysfunction, cytochrome c release, and caspase activation. **(D)** Inflammasome-associated pyroptosis involving NLRP3-associated signaling, caspase-1 activation, gasdermin D processing, and inflammatory mediator production. Melatonin-associated attenuation of these injury-related processes has been reported in selected experimental models. Lipid peroxidation alone does not establish ferroptosis, and inflammasome activation alone does not demonstrate pyroptotic cell death.

#### 4.3.2 Ferroptosis: lipid peroxidation, iron dependence, and evidence criteria

Ferroptosis is an iron-dependent form of regulated cell death driven by uncontrolled phospholipid peroxidation (Jiang et al., 2021). Importantly, increased lipid peroxidation or reduced glutathione peroxidase 4 (GPX4) expression alone is not sufficient to establish ferroptotic cell death. In this review, evidence is considered strongly supportive of ferroptosis when cell injury or death is accompanied by iron dependence and phospholipid peroxidation and is prevented by ferroptosis-selective interventions, such as ferrostatin-1, liproxstatin-1, iron chelation, or appropriate genetic manipulation of ferroptosis-regulatory machinery. Changes in GPX4, solute carrier family 7 member 11 (SLC7A11), glutathione, acyl-CoA synthetase long-chain family member 4 (ACSL4), labile iron, or lipid-peroxidation markers are regarded as supportive components rather than individually diagnostic criteria.

This distinction is particularly important in spermatozoa. Their polyunsaturated-fatty-acid-rich membranes are physiologically required for membrane fluidity, motility, and fertilization but are intrinsically susceptible to oxidative damage (Lenzi et al., 1996). Therefore, lipid peroxidation in mature spermatozoa should not automatically be labeled ferroptosis. Mature sperm have limited transcriptional and translational activity and may not reproduce all canonical ferroptotic features characterized in nucleated cells. Mitochondrial ROS may increase ferroptotic susceptibility by influencing phospholipid oxidation, iron–sulfur proteins, NADPH-dependent antioxidant capacity, and lipid-radical propagation, although mitochondrial involvement varies with the initiating stimulus and metabolic state (Battaglia et al., 2020; Qiu et al., 2024; Bucarey et al., 2026). Oxidation of cardiolipin and other mitochondrial phospholipids may disrupt respiratory-chain organization, increase electron leakage, and create a feed-forward cycle between membrane damage and ROS production. Importantly, mitochondria are not obligatory for every ferroptotic model (Battaglia et al., 2020). Direct demonstration of mitochondrial cardiolipin oxidation and mitochondrial phospholipid-peroxide formation during testicular ferroptosis remains limited (Battaglia et al., 2020; Yang et al., 2022; Cai et al., 2025). Redox-active ferrous iron promotes lipid-peroxide formation. Ferroptotic susceptibility also depends on transferrin uptake, ferritin storage, and NCOA4-mediated ferritinophagy, iron export, heme and iron–sulfur metabolism, and mitochondrial iron handling. Transferrin-receptor-dependent ferroptosis has been linked to phthalate-associated blood–testis barrier injury (Zhao et al., 2023), but mitochondrial labile iron, ferritin, and iron–sulfur integrity remain poorly defined in reproductive cells (Yang et al., 2022). nuclear receptor coactivator 4 (NCOA4) regulation by melatonin has been reported only in non-reproductive macrophages (Xu et al., 2024).

GPX4 uses glutathione to reduce phospholipid hydroperoxides and is essential for spermatogenesis (Ingold et al., 2018; Jiang et al., 2021; Zhao et al., 2023; Wu et al., 2024). In reproductive injury models, melatonin exposure was associated with G6PD/glutathione-related protection during HT-2 toxin exposure (Ma et al., 2023a), with reduced mitochondrial dysfunction and ferroptosis-related injury together with MT2/AKT-associated signaling in swine testicular cells (Zhao et al., 2024), and with altered autophagy/ferroptosis-related responses in heat-stressed mouse spermatocytes (Lei et al., 2025). These studies support involvement of glutathione-, GPX4-, and MT2/AKT-related mechanisms in selected contexts but do not establish a universal ferroptosis-suppressive pathway (**Figure 4B**). ferroptosis suppressor protein 1 (FSP1)–coenzyme Q (CoQ) and mitochondrial dihydroorotate dehydrogenase (DHODH) provide GPX4-independent defense (Gao et al., 2022), yet neither has been directly validated as a melatonin target in reproductive cells.

#### 4.3.3 Pyroptosis: gasdermin-dependent lytic cell death and blood–testis barrier consequences

Pyroptosis is a gasdermin-dependent form of lytic regulated cell death and should be distinguished from inflammasome activation alone. Increased NLRP3 expression, caspase-1 activity, interleukin-1β, or interleukin-18 indicates inflammasome-associated signaling but does not by itself demonstrate pyroptotic cell death. Stronger evidence for canonical pyroptosis requires caspase-1-dependent gasdermin D (GSDMD) cleavage, gasdermin pore formation or activity, and a compatible lytic phenotype such as membrane permeabilization, cell swelling, propidium-iodide uptake, or lactate dehydrogenase release. Genetic or pharmacological disruption of GSDMD or the relevant inflammatory caspase, together with rescue of membrane integrity or cell viability, provides stronger causal evidence. Mitochondrial ROS, oxidized mitochondrial DNA, ATP release, ionic flux, and mitochondrial depolarization may contribute to inflammasome activation in selected contexts (Bours et al., 2006; Shimada et al., 2012; Iyer et al., 2013), but these signals should not be interpreted as a single obligatory mitochondrial pathway.

GSDMD pores contribute to membrane permeabilization by promoting potassium efflux, osmotic water influx, cytokine release, and ultimately lytic cell injury. If GSDMD-dependent pore formation occurs in Sertoli cells, it could theoretically compromise blood–testis barrier integrity through cell swelling or loss, disruption of ionic and osmotic homeostasis, release of IL-1β and IL-18, remodeling of actin and junctional complexes, recruitment of inflammatory cells, and impaired support of developing germ cells. In porcine Sertoli cells, NLRP3 inhibition improved age-related tight-junction impairment (Xiao et al., 2023), supporting an association between inflammasome activity and barrier dysfunction but not demonstrating that GSDMD pore formation directly disrupts the blood–testis barrier. Direct genetic, pharmacological, and functional evidence is therefore still required to establish this mechanism (Samaha and Nour, 2024).

Melatonin alleviated testicular pyroptosis caused by 1,2-dichloroethane in a toxicant-specific piRNA/IRF7 model (Zhang B. et al., 2024). While some studies suggest anti-pyroptotic effects in the eye and fish intestine (Miao et al., 2022; Ye et al., 2022) and even in mammalian testes, they do not provide direct evidence. Plausible mechanisms for reproductive function may include a decreased production of pathological mitochondrial reactive oxygen species (ROS), preservation of mitochondrial membrane integrity, decreased release of mitochondrial DNA, inhibition of NF-κB-dependent priming, inhibition of NLRP3 assembly or caspase-1 activation, preservation of mitophagic flux, and the metabolic and junctional integrity of Sertoli cells. Current evidence does not identify a dominant step, and direct inhibition of cardiolipin-dependent NLRP3 recruitment or GSDMD pore formation by melatonin remains unproven (**Figure 4D**).

#### 4.3.4 Crosstalk among regulated cell-death pathways and evidence boundaries

Apoptosis, ferroptosis, and pyroptosis are mechanistically distinct, but they may coexist or occur sequentially during severe male reproductive injury. Mitochondrial depolarization and outer-membrane permeabilization favor cytochrome c release and caspase-dependent apoptosis, whereas iron dysregulation, glutathione depletion, GPX4 impairment, and phospholipid peroxidation increase ferroptotic susceptibility (Shaha et al., 2010; Tait and Green, 2013; Battaglia et al., 2020; H and B, 2020; Han et al., 2020; Jiang et al., 2021; Yang et al., 2022; Zhao et al., 2023; Fang et al., 2024; Qiu et al., 2024; Wu et al., 2024; Xu et al., 2024; Yuan et al., 2024; Cai et al., 2025; Bucarey et al., 2026). Meanwhile, mitochondrial reactive oxygen species, ATP release, oxidized mitochondrial DNA, cardiolipin redistribution and ionic imbalance can facilitate the activation of the inflammasome and subsequent GSDMD-mediated pyroptosis (Bours et al., 2006; Shimada et al., 2012; Iyer et al., 2013; Oduro et al., 2022; Samaha and Nour, 2024; Zhang W. et al., 2025). Impaired mitophagy and NADPH depletion can connect these pathways through membrane damage and inflammatory priming. Convergence of mitophagy and redox stress is insufficient to establish convergence of the execution programs (**Figure 4A**).

This distinction is particularly important in the testis because multiple cell populations occupy adjacent compartments. Increased cleaved caspase-3, reduced GPX4, and increased NLRP3, caspase-1, or GSDMD detected in whole-testis homogenates may arise from different cell types rather than from simultaneous activation of several death programs within the same cell. Germ-cell apoptosis, ferroptosis-related injury in Sertoli or germ cells, inflammasome activation or pyroptosis in Leydig or immune cells, and secondary inflammatory responses may coexist at the tissue level without sharing a single temporal sequence. Therefore, in the absence of cell-resolved and time-resolved evidence, co-detection of pathway-associated markers should be interpreted as coexistence or potential crosstalk rather than as proof of sequential execution within the same cell.

Reliable classification of regulated cell death should integrate compatible morphology, pathway-specific biochemical evidence, pharmacological and/or genetic perturbation, rescue experiments, and, where relevant, time-resolved functional outcomes. Spatial imaging, single-cell analysis, cell-specific genetic models, and organotypic testicular systems will be particularly useful for distinguishing cell-autonomous death from secondary injury propagated by cytokines, oxidized lipids, or mitochondrial damage-associated signals. Evidence for melatonin is uneven. Attenuation of mitochondrial dysfunction and apoptosis is supported in several reproductive models (Espino et al., 2011; Li et al., 2018; Li et al., 2020; Sun et al., 2022; Chen et al., 2024; Liu et al., 2025; Niu et al., 2025), whereas ferroptosis-related evidence is restricted to toxin-, heat-, or experimentally induced models (Ma et al., 2023a; Zhao et al., 2024; Lei et al., 2025), and direct reproductive evidence for pyroptosis inhibition is largely limited to the 1,2-dichloroethane model (Zhang B. et al., 2024). Direct regulation by melatonin of mitochondrial labile iron, NCOA4-dependent ferritinophagy, FSP1–CoQ or DHODH defense, cardiolipin- or oxidized-mitochondrial DNA-dependent NLRP3 assembly, and GSDMD-mediated blood–testis barrier disruption remains insufficiently demonstrated (Shimada et al., 2012; Iyer et al., 2013; Gao et al., 2022; Yang et al., 2022; Xiao et al., 2023; Samaha and Nour, 2024; Xu et al., 2024). Thus, melatonin should be regarded as a context-dependent modulator of shared stress networks rather than a universal inhibitor of all regulated cell-death pathways (**Figure 3D**).

#### 4.4 Integrative synthesis: a proposed initiation–amplification–progression framework

Taken together, the reviewed evidence can be organized into three overlapping and potentially reversible phases rather than a fixed linear pathway (**Figure 3**). In the proposed framework, initiation refers to cell-specific disturbances produced by toxicants, metabolic stress, inflammation, heat, ischemia–reperfusion, aging, circadian disruption, or cryopreservation, including excessive or mislocalized ROS, endocrine or receptor disturbance, DNA damage, and junctional injury. Amplification refers to conditions in which mitochondrial dysfunction, impaired quality control, inflammatory signaling, mitochondrial damage-associated molecular patterns, and lipid peroxidation reinforce one another. Progression refers to persistent or insufficiently resolved injury associated with impaired Leydig-cell steroidogenesis, Sertoli-cell metabolic and barrier support, germ-cell survival and differentiation, and sperm function. Importantly, these phases represent an interpretation of the available literature rather than a universally demonstrated temporal sequence. Mitochondrial dysfunction may be primary, secondary, or part of a bidirectional feedback loop depending on the initiating insult. Within this framework, melatonin is best viewed as a context-dependent modifier that may act at different points of the network rather than as a universal inhibitor of a single upstream pathway. The strength and limitations of evidence supporting individual components of this model are summarized in **Table 4**.

**Table 4:** Evidence hierarchy for ROS-driven pathogenic networks.

Stage/mechanism	Representative context	Evidence interpretation	Main limitation	References
ROS imbalance	Toxicant, metabolic, heat, or inflammatory stress	Mitochondrial and non-mitochondrial ROS can initiate injury	ROS also support normal reproductive signaling	(Brookes et al., 2004; Murphy, 2009; Galluzzi et al., 2012; Nunnari and Suomalainen, 2012; Bejarano et al., 2014; Chandel, 2015; Bravo et al., 2024; Pundkar et al., 2025)
Bioenergetic protection	Mitochondrial injury models	Melatonin preserves membrane potential, respiration, or ATP in selected models.	Exposure and receptor independence are rarely measured	(Martín et al., 2000; Martín et al., 2002; Reiter et al., 2003; Jou et al., 2007; Hardeland et al., 2009; Galano et al., 2011; Zhao et al., 2024; Liu et al., 2025; Niu et al., 2025; Wen et al., 2025)
Sirtuin/NRF2 adaptation	Toxicant or metabolic injury	SIRT1-, SIRT3-, PGC-1α-, FOXO1-, or NRF2-associated protection	No universal cascade across species or insults	(Kumar et al., 2021b; Ma et al., 2021; Zhang et al., 2022; Habiba et al., 2023; Liu J. et al., 2024; Liu et al., 2025)
Dynamics and mitophagy	Heat, cadmium, or cryopreservation	Melatonin may normalize mitochondrial quality-control flux	Static markers do not prove functional flux	(Hu et al., 2016; Reiter et al., 2017; Jiang et al., 2022; Lu et al., 2022; Yi et al., 2022; Lan et al., 2025; Wen et al., 2025; Ma et al., 2026)
NF-κB amplification	LPS-associated inflammation	Reduced TLR4/MyD88/NF-κB or SIRT1/NF-κB signaling	Mitochondrial repair may not be the initiating event	(Kumar et al., 2021a; Feng et al., 2022)
NLRP3 activation	Mitochondrial danger signals and inflammasome models	Strong general mechanism with selected reproductive support	Direct mtDNA/cardiolipin causality in reproductive cells is limited	(Bours et al., 2006; West et al., 2011; Shimada et al., 2012; Iyer et al., 2013; Weinberg et al., 2015; Hong et al., 2021; Oduro et al., 2022; Almarzouq and Al-Maghrebi, 2023; Wang et al., 2023; Ruan et al., 2024; Elhessy et al., 2025; Wei et al., 2025; Zhang X. et al., 2025; Kamel and Hussein, 2026)
Apoptosis	Toxicant, heat, metabolic, or sperm injury	Repeated reproductive evidence for mitochondria-associated apoptosis	Molecular rescue does not equal restored fertility	(Espino et al., 2011; Li et al., 2018; H and B, 2020; Han et al., 2020; Li et al., 2020; Sun et al., 2022; Chen et al., 2024; Fang et al., 2024; Yuan et al., 2024; Liu et al., 2025; Niu et al., 2025)
Ferroptosis	Toxin, phthalate, heat, or experimental injury	GPX4/glutathione- and MT2/AKT-related protection in selected models	Sperm lipid peroxidation is not automatically ferroptosis	(Ingold et al., 2018; Battaglia et al., 2020; Jiang et al., 2021; Yang et al., 2022; Ma et al., 2023a; Zhao et al., 2023; Qiu et al., 2024; Wu et al., 2024; Xu et al., 2024; Cai et al., 2025; Lei et al., 2025; Bucarey et al., 2026)
Pyroptosis	Obesity, toxicant, heat, aging, or ischemia	NLRP3/caspase-1/GSDMD pathways occur in selected testicular models	Cell identity and sequence vary by model	(Hong et al., 2021; Almarzouq and Al-Maghrebi, 2023; Wang et al., 2023; Xiao et al., 2023; Liu H. et al., 2024; Samaha and Nour, 2024; Zhang B. et al., 2024; Elhessy et al., 2025; Dai et al., 2026; Kamel and Hussein, 2026)
PANoptosis	Coexisting death-pathway markers	Useful integrative hypothesis	Co-occurrence does not prove same-cell execution	(Ye et al., 2022)

## 5 Translational boundaries and future perspectives

Most evidence linking melatonin with redox, mitochondrial, inflammatory, and regulated cell-death-associated responses in male reproduction derives from rodents, livestock, cultured reproductive cells, or acute experimental injury models. These studies establish biological plausibility and model-specific mechanistic support rather than clinical efficacy. Human *in vivo* observations, human ex vivo sperm studies, animal models, reproductive-cell cultures, and indirect non-reproductive mechanistic studies are therefore considered distinct evidence levels and should not be interpreted interchangeably.

### 5.1 Human evidence and clinical trial considerations

#### 5.1.1 Current human evidence and translational endpoint hierarchy

Human evidence remains limited and heterogeneous and should be separated according to experimental context. Observations from testes of infertile men suggest local melatonin-related biological activity (Rossi et al., 2014), but they are observational and do not establish treatment causality. In isolated human sperm, melatonin was associated with receptor/ERK-dependent attenuation of apoptosis-related changes (Espino et al., 2011) and reduced oxidative stress-induced DNA damage ex vivo (Bejarano et al., 2014). A human supplementation study reported changes in inflammatory and oxidative-stress-related testicular markers in men with abnormal spermatogenesis of unknown cause (Riviere et al., 2020), but these findings do not demonstrate recovery of spermatogenesis, improved natural conception, clinical pregnancy, or live birth. Likewise, associations between sleep disturbance and semen quality (Jensen et al., 2013) do not establish that reduced melatonin mediates the relationship or that melatonin supplementation would reverse it.

Adequately powered, male-specific, placebo-controlled trials demonstrating improvement in natural conception, clinical pregnancy, live birth, durable recovery of spermatogenesis, or offspring health are currently lacking. Human evidence instead derives primarily from testicular observations, ex vivo sperm studies, cryopreservation experiments, and heterogeneous assisted-reproduction settings (Espino et al., 2011; Bejarano et al., 2014; Zetner et al., 2016; Hu et al., 2020; Riviere et al., 2020; Menczel Schrire et al., 2022). Systemic administration, direct semen supplementation, cryopreservation additives, and interventions involving the female partner are biologically and pharmacokinetically distinct and should not be combined when interpreting efficacy. To avoid treating findings from different experimental levels as equivalent, representative evidence is stratified by species, biological material, and experimental system in **Table 5**. Melatonin should therefore remain a context-dependent experimental reproductive modulator rather than an established fertility therapy.

**Table 5:** Evidence for melatonin in male reproductive biology by species and experimental system.

Evidence category	Species/model	Experimental system	Main evidence	References
Human	Men with altered spermatogenesis/infertile testes	Supplementation/tissue observation	Reduced oxidative and inflammatory alterations	(Rossi et al., 2014; Riviere et al., 2020)
Human ex vivo	Human sperm/semen	Isolated sperm/cryopreservation	Reduced apoptosis-related changes and DNA damage	(Espino et al., 2011; Bejarano et al., 2014; Al Bijou et al., 2025)
Rodent	Mouse testis, Leydig and germ cells	*In vivo*/cell culture	Steroidogenic, redox and apoptosis-related responses	(Li et al., 2018; Gao et al., 2019; Li et al., 2020; Sun et al., 2022; Zhang et al., 2022; Habiba et al., 2023; Liu et al., 2025)
Livestock	Calf, sheep and swine reproductive cells	Cell culture	Inflammatory, metabolic and AKT-related responses	(Deng et al., 2022; Feng et al., 2022; Sun et al., 2022; Zhao et al., 2024)
Animal *in vivo*	Toxicant/inflammatory testicular models	*In vivo* injury models	Reduced oxidative, inflammatory, barrier or cell-death injury	(Deng et al., 2020; Zhang B. et al., 2024; Niu et al., 2025)
Ex vivo tissue	Cryopreserved testicular tissue	Tissue preservation	Reduced oxidative stress and PINK1–Parkin-related changes	(Ma et al., 2026)

Clinical interpretation requires an explicit endpoint hierarchy. Changes in ROS, malondialdehyde, cytokines, caspases, mitochondrial markers, or pathway-associated proteins may indicate target engagement but are not equivalent to restored fertility. Translational assessment should distinguish molecular target engagement and reproductive-cell function from semen and endocrine outcomes and, ultimately, from fertilization, pregnancy, live birth, offspring health, and durable reproductive recovery. Because physiological ROS contribute to capacitation and fertilization-related signaling, improvement in oxidative biomarkers does not necessarily imply improvement in sperm function. Human studies therefore require cause-specific patient selection and integration of molecular, semen, endocrine, and clinically meaningful reproductive endpoints.

#### 5.1.2 Clinical trial design: population, dose, timing, and duration

Patient heterogeneity is a major determinant of trial design. Oxidative, metabolic, inflammatory, circadian, seminal-leukocyte, sperm-membrane, and selected testicular phenotypes may differ in responsiveness, whereas major chromosomal abnormalities, Y-chromosome deletions, congenital absence of the vas deferens, complete obstruction, and irreversible germ-cell loss are unlikely to be corrected by melatonin (Agarwal et al., 2021; Minhas et al., 2025; Zhao et al., 2025). Trials should use etiological and biomarker-based stratification rather than enrolling all men with infertility as one group. Baseline assessment should include semen phenotype, sperm DNA integrity, selected redox and inflammatory markers, reproductive hormones, metabolic and sleep status, age, and medication use (Pasqualotto et al., 2000; Jensen et al., 2013; Ribas-Maynou and Yeste, 2020).

Dose, formulation, administration time, and treatment duration remain uncertain. Lower exposures may engage specific receptors or circadian signaling. Higher exposures can lead to chemical scavenging or off-target effects. Scale-up of animal doses to human is not feasible without considering pharmacokinetics. Human absorption, bioavailability, and first-pass metabolism vary with different dosage forms and administration routes (Harpsøe et al., 2015; Zetner et al., 2016). Chronopharmacology is a necessary consideration, as there are many variables which operate in a rhythmic fashion, including melatonin secretion, testosterone, testicular clocks, metabolism and mitochondrial function (Bremner et al., 1983; Alvarez et al., 2008; Koike et al., 2012; Jensen et al., 2013; Peek et al., 2013; Partch et al., 2014; Baburski et al., 2015; Claustrat and Leston, 2015; Tordjman et al., 2017; de Goede et al., 2018; Meneses-Santos et al., 2018; Coelho et al., 2019). Light–dark conditions, as well as administration and sampling times, baseline circadian phase, formulations, and plasma exposure should be reported. Where feasible, seminal or tissue melatonin concentrations should also be reported.

Treatment duration should match the biological target. Ex vivo sperm protection may happen within hours (Espino et al., 2011; Bejarano et al., 2014), while spermatogenesis requires assessment across an appropriate time frame. Short-term exposure may alter biomarkers, while long-term exposure could alter endocrine or circadian function. The response to immediate- and sustained-release formulations should be evaluated independently (Harpsøe et al., 2015; Zetner et al., 2016). Dose-escalation studies should define exposure limits and systematically assess reproductive and systemic adverse effects. Evidence of impaired capacitation, endocrine disturbance, excessive sedation, or other clinically relevant adverse effects should inform predefined dose-limiting or discontinuation criteria. Persistence after discontinuation may be attributed to an antioxidant effect.

Randomized placebo-controlled trials are the most appropriate for future studies. Participants should be separated into etiological subgroups and phenotypic biomarker groups and should not be lumped into the male infertility diagnosis. Study parameters should include the formulation, route, dose, timing, duration, adherence, and use of co-administered antioxidants. Primary outcomes would include semen parameters, integrity of sperm DNA, and live birth. However, biomarkers should be used to evaluate target engagement and not assess clinical outcomes.

#### 5.1.3 Safety, endocrine effects, and reproductive monitoring

Despite safety concerns with regard to long-term use, melatonin shows short-term benefits for adults with sleep disorders (Menczel Schrire et al., 2022). Potential risks include somnolence, altered sleep patterns, drug and supplement interactions, variability in melatonin, and an excessive amount of antioxidants. Long-term studies also need to evaluate the effects of melatonin on fertility. For these studies, researchers need to evaluate testicular volume and semen quality. They need to check for sperm DNA integrity, sexual function, and pregnancy outcomes and congenital abnormalities. Long-term studies also need to evaluate recovery after treatment. The negative effect of melatonin on testosterone production in rooster Leydig cells (Zhu et al., 2022) suggests that a presumed positive effect on the endocrine system of males is likely not the case.

### 5.2 Assisted reproduction and functional fertility outcomes

#### 5.2.1 Semen processing, sperm cryopreservation, and tissue preservation

Adding melatonin at specific steps of semen processing, cryopreservation, thawing, sperm selection, and tissue preservation eliminates some uncertainty regarding systemic pharmacokinetics. Evidence shows that ex vivo human sperm protection is provided by melatonin (Espino et al., 2011; Bejarano et al., 2014) and that melatonin lowered cryopreservation-associated sperm DNA fragmentation in a human study (Al Bijou et al., 2025). Melatonin may improve the preservation of cryopreserved testicular tissue and modulate the ROS/PINK1–Parkin mitophagy axis (Ma et al., 2026), though this cannot be generalized to achieve restoration of spermatogenesis. Given that the damage caused by cryopreservation and the freezing process is oxidative and that excessive antioxidants may suppress capacitation, studies evaluating the integration of sperm motility, mitochondrial membrane potential, DNA integrity, capacitation, the acrosome reaction, fertilization, development of the embryo, pregnancy, and the live birth of offspring are warranted.

#### 5.2.2 Clinical assisted-reproduction outcomes

According to meta-analyses, progress has been made to improve outcomes associated with assisted reproduction, especially those related to fertilization, though the data have low certainty and are of limited relevance to males (Hu et al., 2020; Veiga et al., 2024). Studies have failed to consider male-specific factors. Future studies should detail the treated individual, dosage, timing, formulation, semen phenotype, post-thaw function, fertilization, and embryo development as well as pregnancy, live birth, and safety outcomes. Background antioxidant use, reductive stress, laboratory procedures, and female partner factors should be controlled.

### 5.3 Priority questions for mechanistic and clinical translation

#### 5.3.1 Mitochondrial synthesis, RORα, and cell specificity

Whether male reproductive mitochondria can synthesize melatonin *de novo* is still unclear (He et al., 2016; Suofu et al., 2017; Zhu et al., 2024). This is due to a lack of contamination-controlled organelle purification, isotope tracing, AANAT or ASMT manipulation, cell-specific models, and human validation. RORα should remain classified as a possible downstream or parallel regulator until physiological binding, genetic, and chromatin-occupancy studies separate its effects from MT1/MT2, circadian, and redox signaling (Coelho et al., 2019; Li et al., 2020). Passive diffusion and transporter-mediated uptake should be tested using labelled melatonin, transporter perturbation, kinetic analyses, and organelle-resolved mass spectrometry (Hevia et al., 2015; Reiter et al., 2016; Huo et al., 2017).

Whole-testis measurements obscure cell heterogeneity. Single-cell and spatial omics, cell-specific genetics, and organelle-resolved metabolomics should identify responsive Leydig, Sertoli, germ, immune, stromal, and sperm populations and determine where apoptosis, ferroptosis, and pyroptosis occur. Studies should also define a physiological ROS window and test whether melatonin reduces injury without suppressing steroidogenesis, capacitation, acrosome reaction, or fertilization.

#### 5.3.2 Emerging mechanisms and trial readiness

Dietary and microbial contributions require quantitative studies linking intake or microbial production with circulating, seminal, and testicular exposure and reproductive outcomes (Konopelski and Ufnal, 2018; Salehi et al., 2019; Que et al., 2020; Niu et al., 2024; Zanirate et al., 2026). Intercellular mitochondrial transfer requires lineage tracing to determine whether it occurs in the testis, is enhanced by melatonin, and restores function (Nasoni et al., 2021; Iorio et al., 2024).

Ferroptosis studies should assess labile iron, ferritinophagy, FSP1–CoQ, DHODH, phospholipid composition, and cardiolipin oxidation (Battaglia et al., 2020; Jiang et al., 2021; Yang et al., 2022; Zhao et al., 2023; Qiu et al., 2024; Cai et al., 2025; Bucarey et al., 2026). Studies of pyroptosis should incorporate GSDMD-focused genetic or pharmacological approaches, membrane-permeability assays, junctional imaging, cytokine analysis or neutralization, and cell-specific models (Hong et al., 2021; Wang et al., 2023; Ruan et al., 2024; Samaha and Nour, 2024; Elhessy et al., 2025; Wei et al., 2025; Zhang X. et al., 2025; Kamel and Hussein, 2026). Contributions from NLRP3, cGAS–STING, TXNIP, mitochondrial DNA, cardiolipin, and ionic flux should be experimentally distinguished rather than collectively attributed to a single melatonin-responsive pathway (Bours et al., 2006; West et al., 2011; Iyer et al., 2013; Weinberg et al., 2015; Oduro et al., 2022; Almarzouq and Al-Maghrebi, 2023). Before efficacy trials are undertaken, pharmacokinetic studies should characterize formulation-dependent absorption, plasma exposure, seminal and testicular distribution, circadian timing, and dose–response relationships (Harpsøe et al., 2015; Zetner et al., 2016). Subsequent trials should incorporate etiological stratification, clinically meaningful reproductive endpoints, target-engagement biomarkers, and systematic assessment of reproductive and long-term safety.

## 6 Conclusions

This critical narrative review supports an evidence-calibrated and cell-specific interpretation of melatonin-responsive stress signaling in male reproduction rather than a universal melatonin pathway. Physiological and pathological ROS should be distinguished, mitochondrial dysfunction may be primary or secondary depending on the initiating insult, and inflammatory and regulated cell-death pathways require mechanism-specific evidence. Within the proposed initiation–amplification–progression framework, melatonin is best considered a context-dependent experimental modulator that may influence receptor-associated signaling, redox balance, mitochondrial function, inflammatory amplification, and cell-death-associated injury at different stages. Importantly, pathway association should not be equated with causality, lipid peroxidation or altered GPX4 should not automatically be equated with ferroptosis, and inflammasome activation should not automatically be equated with pyroptotic cell death. RORα remains a possible downstream or parallel regulator, and autonomous melatonin synthesis in male reproductive mitochondria remains unproven. Most mechanistic evidence is preclinical or ex vivo; therefore, pharmacologically relevant exposure, target engagement, long-term reproductive safety, natural conception, pregnancy, and live-birth outcomes require direct validation in appropriately designed human studies.
