# Supplementary material for: Mitochondrial stress-signaling networks in male reproductive dysfunction: redox, inflammatory, and cell-death integration with context-dependent modulation by melatonin
Source: Front Physiol. 2026 Oct 28;17:1990053. doi: 10.3389/fphys.2026.1990053 (PMC13752977; doi:10.3389/fphys.2026.1990053)
Supplement: Supplementary file 1 [file Table1.docx]

Supplementary Material

# 1 Supplementary Table S1

**Supplementary Table S1. Database-specific search strategy and consolidated evidence selection**

Searches were conducted through 31 July 2026 in PubMed/MEDLINE, Web of Science Core Collection, and Scopus, using the database-specific strategies shown below. No language, species, or study-design restrictions were applied at the database-search stage. During narrative evidence selection, studies published within the preceding 10 years (1 August 2016–31 July 2026) were prioritized for contemporary mechanistic and translational evidence. Earlier seminal or foundational studies were retained when necessary to support historical context, receptor nomenclature, established mechanistic concepts, or landmark experimental findings. The 10-year interval was therefore used as an evidence-prioritization criterion rather than as an absolute date-based exclusion criterion.

| **Database / stage** | **Coverage** | **Database-specific strategy / rule** | **Records / status** |
| --- | --- | --- | --- |
| **PubMed** | Search: 1 Jan 1900–31 Jul 2026 | (melatonin[Title/Abstract] OR "N-acetyl-5-methoxytryptamine"[Title/Abstract]) AND ("male reproduction"[Title/Abstract] OR "male reproductive"[Title/Abstract] OR "male infertility"[Title/Abstract] OR testis[Title/Abstract] OR testes[Title/Abstract] OR testicular[Title/Abstract] OR Leydig[Title/Abstract] OR Sertoli[Title/Abstract] OR spermatogen*[Title/Abstract] OR "germ cell"[Title/Abstract] OR "germ cells"[Title/Abstract] OR sperm*[Title/Abstract] OR semen[Title/Abstract] OR epididym*[Title/Abstract] OR "blood-testis barrier"[Title/Abstract]) AND (mitochondri*[Title/Abstract] OR "oxidative stress"[Title/Abstract] OR redox[Title/Abstract] OR "reactive oxygen species"[Title/Abstract] OR ROS[Title/Abstract] OR inflammat*[Title/Abstract] OR inflammasome[Title/Abstract] OR NLRP3[Title/Abstract] OR apoptosis[Title/Abstract] OR apoptotic[Title/Abstract] OR ferroptosis[Title/Abstract] OR ferroptotic[Title/Abstract] OR pyroptosis[Title/Abstract] OR pyroptotic[Title/Abstract] OR "regulated cell death"[Title/Abstract] OR mitophagy[Title/Abstract] OR autophagy[Title/Abstract] OR "mitochondrial dynamics"[Title/Abstract] OR MT1[Title/Abstract] OR MT2[Title/Abstract] OR MTNR1A[Title/Abstract] OR MTNR1B[Title/Abstract] OR SIRT1[Title/Abstract] OR SIRT3[Title/Abstract] OR NRF2[Title/Abstract] OR "PGC-1alpha"[Title/Abstract] OR circadian[Title/Abstract] OR RORalpha[Title/Abstract] OR AANAT[Title/Abstract] OR ASMT[Title/Abstract] OR GLUT[Title/Abstract] OR PEPT1[Title/Abstract] OR PEPT2[Title/Abstract] OR transporter*[Title/Abstract]) AND ("1900/01/01"[Date - Publication] : "2026/07/31"[Date - Publication]) | **n = 635** |
| **Web of Science Core Collection** | Search: inception–31 Jul 2026 | Topic: (melatonin OR "N-acetyl-5-methoxytryptamine") AND (testis OR testes OR testicular OR Leydig OR Sertoli OR spermatogen* OR sperm* OR semen OR (male NEAR/1 infertil*) OR "blood-testis barrier") AND (mitochondri* OR "oxidative stress" OR "reactive oxygen species" OR redox OR NLRP3 OR ferroptosis OR pyroptosis OR mitophagy) | **n = 2,201** |
| **Scopus** | Search: inception–31 Jul 2026 | TITLE-ABS-KEY((melatonin OR "N-acetyl-5-methoxytryptamine") AND (testis OR testes OR testicular OR leydig OR sertoli OR spermatogen* OR sperm* OR semen OR (male AND infertil*) OR "blood-testis barrier") AND (mitochondri* OR "oxidative stress" OR "reactive oxygen species" OR redox OR NLRP3 OR ferroptosis OR pyroptosis OR mitophagy)) AND PUBYEAR < 2027 | **n = 748** |
| **Consolidated post-cleaning set** | Evidence priority: 1 Aug 2016–31 Jul 2026 | Merge all database records and remove exact/near-duplicate records. Prioritize literature published within the preceding 10 years (1 Aug 2016–31 Jul 2026). Retain a limited number of earlier seminal/foundational studies when essential for historical context, receptor nomenclature, established mechanistic concepts, or landmark evidence. | Total records identified: n = 3,584; retained after de-duplication and relevance screening: n = 314 |
| **Final cited evidence set** | Current revised manuscript | Eligible records were narratively evaluated and supplemented, when necessary, by targeted citation checking. Cited evidence was classified as primary male-reproductive studies, reproductive secondary evidence, or indirect/general mechanistic and background evidence. | **Primary male-reproductive studies: n = 92; reproductive secondary evidence: n = 41; indirect/general background: n = 86; total cited references: n = 219** |

Note. The 10-year interval was used as a literature-prioritization criterion rather than an absolute exclusion rule. Earlier studies were retained selectively when needed for historical context, receptor nomenclature, established mechanistic concepts, or landmark experimental evidence. After merging the three database exports, de-duplication and relevance screening yielded n = 314 candidate records; the final cited evidence set comprised n = 219 references.
